# Characteristics and Models of Moisture Uptake in Fiber-Reinforced Composites: A Topical Review

**DOI:** 10.3390/polym16162265

**Published:** 2024-08-09

**Authors:** Behnaz Hassanpour, Vistasp M. Karbhari

**Affiliations:** 1 Department of Civil Engineering, University of Texas Arlington, Arlington, TX 76006, USA; vkarbhari@uta.edu; 2 Department of Mechanical and Aerospace Engineering, University of Texas Arlington, Arlington, TX 76006, USA

**Keywords:** fiber, resin, composite, interface, degradation, hygrothermal, diffusion, moisture, Fickian, non-Fickian, humidity

## Abstract

Fiber-reinforced composites are commonly exposed to environments associated with moisture and solution, resulting in uptake, which causes changes in the bulk resin, the fiber–matrix interface, and even the fiber itself. Knowledge about uptake behavior and diffusion mechanisms and characteristics are critical to better understanding the response of these materials to environmental exposure faced through service to developing better materials through selection of constituents and to the prediction of long-term durability. This paper reviews aspects of uptake mechanisms and subsequent response, as well as models that describe the sorption process, with the aim of providing a comprehensive understanding of moisture-uptake-related phenomena and characteristics such as uptake rate, diffusion and relaxation/deterioration constants, transitions in regimes, and overall response.

## 1. Introduction

Fiber-reinforced polymer (FRP) composites are widely used in civil infrastructure in addition to their more common application in automotive, marine/naval, and aerospace sectors due to their favorable characteristics, such as being lightweight, their high specific strength and stiffness, and their tailorability. Despite their successful use in a large number of applications, there are still concerns related to the long-term durability of the materials in harsh and changing environmental conditions [1,2,3,4,5]. While FRP composites have potentially enhanced durability compared to conventional materials, they undergo various levels of degradation because of environmental exposure. The response of these materials is dependent on the level of void content, type of exposure, constituents of the composites, and on the fiber–resin interface [6,7,8,9,10,11], their volume fractions, fiber configuration [2], and the process used to fabricate the component, which can result in different levels of cure progression. This, in turn, can result in changes in propensity for moisture uptake and/or environment-induced microcracking. Furthermore, environmental exposure in the field is rarely due to a single isolated environment and is generally a synergistic effect of multiple conditions, which could change and accelerate the degradation process. In addition, most data are collected through short-term laboratory testing, which rarely covers the full range and extent of exposure in the field and resulting deterioration. The effects of moisture and solution through humidity and/or immersion are often the most common in civil infrastructure applications, and these conditions can result in significant mechanical degradation, potentially causing premature failure [12,13]. To ensure the safe design of FRP structures, it is necessary to address the long-term environmental effects on performance characteristics and service life. The degree of vulnerability to degradation depends on the environment’s characteristics and the distinct reactions of each constituent. Although some fibers, such as E-glass and aramid, are susceptible to moisture, others such as carbon and basalt remain unaffected by it. However, both the resin system and the fiber–matrix interphase are vulnerable to various environmental factors, with moisture being a significant concern since it can affect the integrity of the fiber–matrix bond, as well as degrade the constituents.

To predict the service life and durability of FRPs, it is essential to develop a comprehensive understanding of the mechanisms of environmental deterioration and aging. While several environmental exposure drivers exist, such as temperature, cycling, rain, sunshine, UV, etc., a substantial understanding can be gleaned from a closer examination of water’s effects on different components within the system. Polymer-based materials exhibit variable water-absorption capacities, depending on factors such as their chemical composition, formulation, and the ambient humidity and temperature conditions [14,15,16]. Consequently, the extent of absorption is determined not only by the material’s chemical compositions and formulation, but also by the hygrothermal conditions to which they are exposed [17]. Temperature fluctuations and moisture absorption, whether through humidity or solution, induce notable modifications in the physical and chemical attributes of the polymeric matrix, consequently leading to potential degradation in the polymers and at the interface between the polymer and fiber [18]. Moisture-induced changes involve the hydrolysis of the polymer matrix chain and interfacial bond, resulting from the interruption of interchain hydrogen bonds by water molecules. This can cause an increase in intersegmental hydrogen bond length [15], increased chain mobility [19], and even internal void formation accompanying saponification [20]. Physical degradation processes include swelling, plasticization, polymer relaxation, degradation of molecular and network structure, and weakening of the fiber–matrix bond and interphase [9,15,19,21,22,23,24,25,26,27,28,29]. Swelling can lead to microcracking in the weakened resin that has undergone hydrolysis, contributing to further interfacial debonding at the hydrolyzed interface, which may result in accelerated physio-mechanical degradation [27,30,31,32,33,34,35,36,37,38]. The chemical and physical effects resulting from exposure to hygrothermal conditions can alter the properties of polymer matrix composites, with some changes being reversible, while others are irreversible and permanent [39,40]. It is emphasized, however, that reversibility is phenomenological and depends on the details of environmental exposure and material constituents. In some cases, such as when elevated ambient temperatures follow periods of high humidity or immersion, moisture uptake and its effect can be reversed. In other cases, long periods of immersion result in reversible mechanisms not being activated. There is thus a considerable difference between field and laboratory exposure effects, as well as between short-term testing (even under accelerated conditions) and long-term exposure and testing.

While numerous environmental conditions and factors can affect the durability of FRPs, the focus of this review is on generic effects of moisture and solution, highlighting the importance of understanding their transport into the composite and the subsequent effects on the substrates. While the aspects of mechanisms of deterioration and effects of environmental exposure that cause these, as well as that of overall durability, are extremely important, this paper focuses on a topical review of models of diffusion and moisture uptake, thereby providing readers with a nuanced view not just of models, but also of the complex interactions between short- and long-term phenomena that drive the consideration and use of different models and equations that describe them. In addition, the focus is strictly on the phenomena of moisture transport without detail on the chemical reactions and changes in morphology that are also taking place that are best considered through more involved assessments of chemistry.

## 2. Mechanisms and Characteristics Due to Moisture Uptake

In composite materials, the performance of the composite is determined by the properties of the polymer matrix, the reinforcing fibers, and the integrity of the interface between the fiber and polymer matrix. Comprehending the distinct yet interrelated effects of moisture on the matrix, fibers, and fiber–matrix interface is essential for understanding long-term behavior and reliability. The presence of moisture alters bulk polymeric properties through interactions with polymer chains [11,41,42,43,44]. Moisture transport in polymers depends on the presence of molecular-sized voids within the polymer structure and the affinity between the polymer and water. The availability of these voids is influenced by the polymer’s structure, morphology, and crosslink density, while the polymer–water affinity is governed by the presence of hydrogen-bonding sites along the polymer chains, facilitating interactions with water molecules [19].

The process of water transfer within fiber-reinforced polymer composites involves various mechanisms. Water molecules primarily permeate the polymer matrix through diffusion. Additionally, absorbed water moves throughout the composites through capillary action along the fiber–matrix interface and along crack surfaces. This absorbed moisture induces expansion and swelling of the matrix, leading to plasticization and hydrolysis, thus promoting further crack formation and augmenting water diffusion within the composites [45]. To effectively study these interactions, it is important to distinguish between reversible and irreversible changes in the properties of the polymer matrix. This requires clarifying the definition of absorption, adsorption, and desorption, along with an understanding of their role in determining the reversibility of moisture-induced effects [46,47,48,49]. The term “sorption” is a broad term encompassing generic moisture/solution transport mechanisms, typically used when the specific mechanism of uptake cannot be pinpointed.

**Figure 1:**
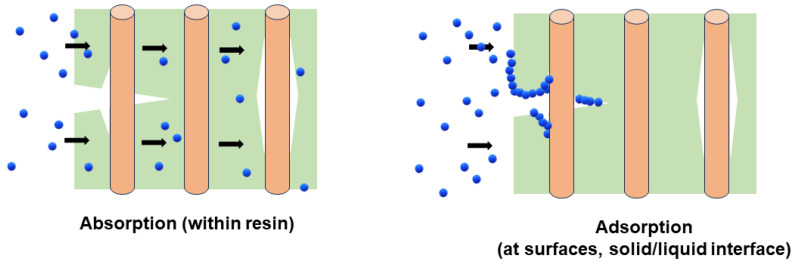
Schematic representation of absorption into the resin and adsorption at surfaces.

As shown in Figure 1, absorption is a process where a liquid or vapor external to a solid (referred to as the sorbate) permeates the bulk solid (referred to as the sorbent). In the case of a composite, absorption involves capillary uptake through voids, microcracks, and interface gaps, filling free space without immediate plasticization or swelling of the resin, and generates very little heat. On the other hand, adsorption takes place when a liquid or vapor external to the solid adheres to the surface of the bulk solid, forming an adsorbed phase that is no longer part of the bulk fluid. This process generates heat (heat of solution) and can result in swelling [50,51]. 

Physisorption and chemisorption are categories within adsorption, with physisorption indicating a weak interaction with the surface and chemisorption involving a strong interaction akin to chemical bonding [52]. The adsorption process is fundamental in the uptake of vapors into porous materials and is also applicable for describing vapor sorption, as well as fluid adsorption onto nonporous solid surfaces [53]. For a polymer free of voids or pores, the uptake of water is mainly an adsorption process. If a polymer contains pores, gaps, air bubbles, or other defects, both absorption and adsorption occur [51]. The pores or pathways must be of sufficient size for molecules to fill the gaps and adsorb/absorb onto the surfaces and into the material [54]. The presence of fibers results in interfacial areas resulting from the bond of the resin to the fiber, and this provides additional potential pathways for uptake. The process by which liquid molecules move randomly and the rate at which they move through the composite’s bulk is known as diffusion [49]. The diffusion of moisture into polymer composites is generally perceived as a combination of absorption and adsorption processes. Liquid molecules penetrate the polymer surface upon direct contact with the composite, then progress through its bulk driven by concentration gradients, filling voids, cracks, and gaps, and subsequently reacting with the bulk polymer [38,55].

Desorption refers to the release of penetrant from the solid, to which it was previously adhered, into the surrounding vacuum or fluid. This occurs if a molecule has sufficient energy to surpass the activation barrier and the binding force. Thermal desorption involves heating the adsorbate to induce the detachment of atoms or molecules from the material. Detection and monitoring of desorption involve observing negative changes in weight, indicating a loss of weight, and complementing the positive values observed during uptake. In composite materials, determination of the level of desorption, which is the release of previously adsorbed and absorbed moisture, helps assess the reversibility of water uptake and understand the effects of interfacial debonding and microcracking resulting from hygrothermal aging. It needs to be considered that desorption is usually difficult to achieve at the same temperature as initial immersion due to the strong interaction between the functional groups of the resin and water molecules, which disassociate only at higher temperatures [16]. For a specific mode of adsorption or absorption, it is assumed that the same mechanism is responsible for both the uptake and desorption, leading to the utilization of the same models for both phenomena. However, it is challenging to conclusively state that the desorption process is a complete reversal of the adsorption or absorption process. Studies indicate that sorption is not entirely reversible, and the diffusion coefficients, while generally assumed to be a single value for each temperature, may not be identical between the uptake and loss of a sorbate [25,26,56,57,58]. Thus, while the term “uptake” is generally assumed to represent the amount of solution that enters the composites, the level could be a combination of the increase in mass due to the sorption of water and the loss in mass due to mechanisms of desorption and loss of lower-molecular-weight species that leach out into the solution. The mass determined through gravimetric means, as commonly used to measure “uptake”, is then the result of the competition between these mechanisms.

The exposure to moisture can result in reversible and irreversible changes, many of which are dependent on the time of exposure, temperature, and the state/type of water. Two types of water are involved in the absorption process. Free/unbound water and bound water [25]. Free water typically is the water that fills the matrix microcracks and voids without any chemical reaction with the polymer. This type of water consists of retained water molecules that can be easily removed through thermal desorption at lower temperatures. On the other hand, bound water is chemically bonded to specific sites in the resin, such as the hydroxyl groups in the resin network. This type of water contains retained water molecules that are more difficult to remove by thermal desorption and require exposure to higher temperature levels to be removed from the resin network [59]. In the early stages of hygrothermal aging, especially during brief periods of lower temperature exposure, aging effects (such as softening), and plasticization may potentially be reversible through drying [60,61]. This reversibility is achievable if free water is involved, as it can be extracted without causing chemical reactions. Thus, reversible changes occur when moisture absorption by fiber-reinforced polymer composites (FRPs) temporarily reduces the physical properties of both the fiber–resin interface and resin. Once the free water undergoes desorption, the performance can return to its original state [60,62]. However, prolonged exposure to the environment, particularly at high temperatures, results in irreversible and permanent alterations in properties. When the resin in composites chemically reacts with bound water it can lead to hydrolysis, causing a plasticizing effect and reduction in properties. This may result in considerable damage, with desorption unable to completely reverse the absorption process. Irreversible changes can occur due to solution-induced degradation, micro-cavitation damage, deterioration of the bond between fiber and resin, and deterioration of the interface layer structure. This indicates that performance cannot be restored after removal of the solvent with irreversible degradation of the matrix, especially at temperatures well above the glass transition temperature (*T_g_*) [27].

The glass transition temperature (*T_g_*) holds great significance as a fundamental thermophysical property of polymers and fiber-reinforced polymer matrix composites. The glass transition temperature is a characteristic temperature range at which an amorphous material undergoes a transformation from a rigid, brittle glassy state to a more viscous, rubbery state. It sets the upper bound for the use temperature of the material system and enables the characterization of both the cure and molecular state of the material since it depends intrinsically on chain extension and crosslinking, both of which increase *T_g_*. As a parameter, it is also strongly dependent on chemical composition, phase morphology, and the presence of water within the polymer and hence serves to identify critical changes, both reversible and irreversible [63].

Moisture desorption is commonly performed to gauge the extent of reversibility in water absorption and to determine the potential effects of chemical and structural changes, interfacial debonding, and microcracking [64]. Desorption generally takes longer than absorption, and materials that have not reached a state of full polymerization might never completely dry because water molecules are chemically bonded to the resin at specific sites. Thus, to eliminate all water, heating above the glass transition temperature (*T_g_*) is likely necessary, which causes deterioration [55,65].

Table 1 outlines degradation mechanisms, classifying them as reversible or irreversible in terms of chemical, physical, and physio-mechanical aspects. It is important to highlight that reversible processes can never achieve complete reversibility because the rearrangement of molecules inevitably results in an increased entropy of the system. Hydrolysis, typically regarded as irreversible [27], might exhibit some reversible traits through condensation or hydration reactions affecting hydrolyzed bonds [32,39,66,67,68].

While there is a significant body of literature on the effect of moisture on resins and composites and of the viability, or lack thereof, of the Fickian diffusion model, there is a lack of a comprehensive review of uptake phenomena, the resulting mechanisms, and a range of models that would enable the reader to assess the suitability of models to best describe various regimes of uptake. This paper discusses that specific need.

**Figure 2:**
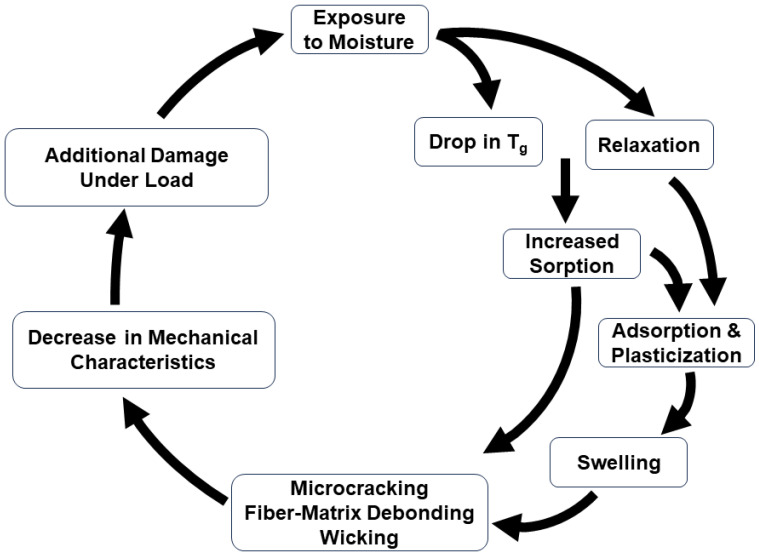
Schematic showing first-order effects of sorption.

Moisture sorption in a polymer matrix can occur through mechanisms of water dissolution within the polymer network and sorption in microscopic voids within the polymer matrix, contributing to the excess free volume in the structure and the formation of hydrogen bonds between water and the hydrophilic groups in the polymer [69]. Given the polar nature of water molecules, they bind to the polymer network through interactions, such as firmly bound direct hydrogen bonds, multi-site interactions, dipole–dipole interactions, and van der Waals forces [70,71]. First-order hydrogen bonding involves water molecules bonding to a strongly polar region of the polymer network through its oxygen or hydrogen atoms. Dipole–dipole interactions are like hydrogen bonds, with lower dissociation energies. Van der Waals forces are characterized by very low dissociation energies. The polar characteristics of water molecules cause disruptions through these reactions, particularly disrupting Van der Waals bonds among polymer chains disturbing the existing interchain hydrogen bonds [71,72,73,74]. These lead to plasticization, enhancing chain segmental mobility [72,75] and swelling due to volumetric expansion as the interchain bond length extends [15,29,76,77], which can potentially result in a decrease in the glass transition temperature, *T_g_*, and mechanical strength characteristics [19]. In addition, the accumulation of water molecules at polar sites on the polymer can further influence the material properties. The first-order effects of sorption, on a composite, are shown in Figure 2. Due to the tendency of water molecules to preferentially form hydrogen bonds with each other rather than with dissimilar polar sites on a polymer, multiple water molecules may accumulate at a single polar site through progressive hydrogen bonding, ultimately resulting in the formation of water clusters [62,78,79,80,81,82]. As water molecules disrupt interchain bonding through the formation of hydrogen bonds and the creation of water clusters, the distance between interchain bonds expands, leading to changes in the overall dimensions of the material [15,83].

The plasticization process is predominantly reversible, i.e., removing water leads to a subsequent *T_g_* increase when transitioning from a “wet” state to a “dry” state [25]. The reversibility suggests that the polymer resin’s mobility is enhanced by the presence of water due to weak interactions [19,84,85]. Water molecules that form multi-site interconnective bond complexes, however, do not contribute significantly to resin plasticization. Instead, these complexes create bridges between chain segments, leading to secondary crosslinking (pseudo crosslinking), which increases *T_g_* [25]. An increase in exposure temperature contributes to a higher amount of multi-site interconnective bond water [25,84], thereby leading to higher *T_g_* with increasing immersion temperatures.

Swelling resulting from water sorption increases relaxation and alters the network structure. As the polymer reacts to the swelling stress from absorbed moisture and dissipates the induced swelling stress initiated by plasticization, the network becomes more accessible to additional water [65]. The change in the polymer structure can become permanent and irreversible under certain conditions, particularly when the polymer undergoes chemical or physical aging. Physical aging occurs when polymer networks, upon cooling into a glassy state, remain in a nonequilibrium condition characterized by excess free volume or enthalpy. This state gradually diminishes over time as the polymer chains attempt to achieve a more stable, thermodynamic equilibrium. This phenomenon involves a gradual decrease in free volume and/or enthalpy, leading to adjustment in the morphology of the polymer chains towards thermodynamic equilibrium [86,87]. The presence of water within the epoxy accelerates the physical aging process due to plasticization, where water increases macromolecular chain mobility, resulting in a faster kinetic rate of physical aging [88]. Chemical aging, on the other hand, occurs when the polymer structure undergoes a chemical reaction, leading to the formation of aged polymer species [86,89]. Residual stresses developed within the polymer network during curing and subsequent cooling may undergo relaxation during the process of physical aging as a means of achieving equilibrium to balance stress disparities between areas with lower and higher crosslink density [86]. The presence of moisture further impacts this process by facilitating stress relaxation through increased chain mobility, thus altering the distribution and intensity of residual stresses. These forces arise due to the thermodynamic nonequilibrium of the glassy network and the internal stresses formed during cooling from higher temperatures. The contraction or shrinkage occurs predominantly in regions with higher crosslink density, where the polymer chains are more tightly bound and thus exhibit a higher tendency to shrink towards their centers of force [86]. With the aging of the polymer, highly crosslinked regions ease towards a central nodular structure due to contractive forces developed during crosslinking [86]. As regions of lower density undergo physical aging, they become denser and consequently create higher levels of tortuosity in the water path. While both aging processes can initially result in an increase in *T_g_*, the processes can also lead to degradation, where the polymer experiences a subsequent decrease in *T_g_* [89].

Short-term plasticization can progress over time by hydrolysis, which causes chemical degradation, particularly of functional groups such as esters and ethers in polymers [43,90,91]. Alkaline hydrolysis, for instance, occurs when OH^−^ reacts with ester bonds, which are the weakest elements in the polymer’s chemical structure. This reaction results in the formation of additional polar hydroxyl groups, potential chain scission, and exposure-related microcracking, and saponification, ultimately resulting in the fracture of the polymer molecular chain and leaching of lower-molecular-weight species (LMWS), with network linkages being attacked with increased exposure and moisture uptake [92,93,94,95]. When the polymer is exposed to water, degradation products with low molecular weight formed due to mechanisms such as chain scission may potentially diffuse out of the polymer matrix, particularly at elevated temperatures. This process can result in irreversible stiffening and embrittlement of the material [96,97,98,99]. Likewise, any remaining LMWS from the initial polymerization reaction and curing process may be driven out at higher temperatures, leading to an apparent weight loss in the polymer [100]. This phenomenon is evidenced by residual post-cure, accompanied by weight loss and some apparent increase in the glass transition temperature, followed by further degradation through chain scission [101]. Other LMWS components, such as plasticizers, mold release agents, and crosslinking agents, may also be desorbed [100,102].

While matrix degradation due to moisture is significant, the fibers themselves are also susceptible to moisture-related damage, impacting the overall integrity of polymer composites. Fibers play a critical role in bearing the loads in polymer composites, with the overall mechanical properties of the composite primarily reliant on fiber performance. Carbon and Basalt fibers show resistance to moisture, while glass and aramid fibers are prone to potential damage while exposed to moisture. Moisture attack on these fibers can lead to chemical degradation, particularly in glass fibers, even before interfacial deterioration occurs [103,104].

**Figure 3:**
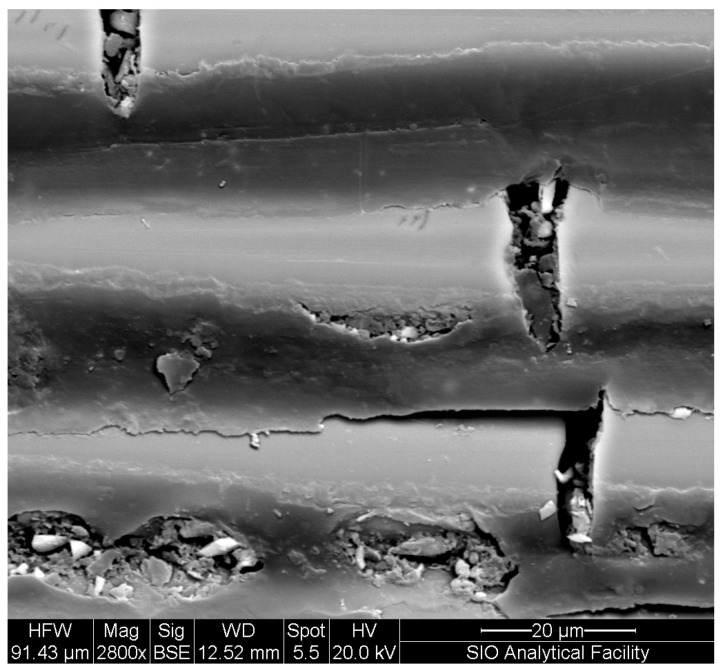
SEM of E-Glass/Vinyl ester showing fiber level deterioration.

The phenomenon of stress corrosion cracking or, more generally, moisture-induced failure in glass fibers and glass fiber composites is recognized in both surface chemistry and fracture mechanics [104]. During the hygrothermal aging process, surface pitting and nanoscale voids can be observed on the glass fiber surface along with premature fiber failure (Figure 3). Initially, there is an induction period necessary for water to attain sufficient mobility on the glass surface [104]. Subsequently, water molecules interact with the silicon–oxide bonds near the glass surface, causing fiber weakening along with pitting and cracking [104]. This process occurs notably when moisture absorption is high, enabling water molecules to move freely on the glass surface and potentially initiate the hydrolysis reaction between silicon–oxide and water [105].

H_2_O + Si-O-Si → Si-OH + HO-Si (1)

This reaction occurs in three phases: adsorption, reaction, and separation. Adsorption is defined as a weak interaction between water molecules and Si-O bonds [105,106].

With glass fibers, degradation initiates as moisture draws out ions from the fiber, thus altering its structure. These ions react with water to form bases, which corrode and pit the fiber surface, creating flaws that significantly weaken strength and may cause premature fracture and failure of the fibers. Direct contact with basic solutions weakens glass fibers, leading to pitting, cracking, and leaching. This can result in rapid loss of the fiber’s core and reactivity of the outer sheath, thereby accelerating the leaching process [20], although the introduction of metal ions in glass has been reported to mitigate degradation. Additionally, the hydrolysis reaction exhibits partial reversibility, with metal hydroxyls potentially forming a metal oxide layer on the fiber surface through condensation reactions or hydrations [67,107]. Therefore, employing a protective coating on the glass fiber surface is crucial to isolate the reinforcement from moisture and simultaneously enhance interfacial adhesion [34,68,107].

Aramid fibers absorb moisture, which can lead to accelerated fibrillation where a thin layer of fibrils is stripped from the fiber surface due to cohesive fiber failure. Solutions like sodium hydroxide and hydrochloric acid are known to significantly speed up hydrolysis, particularly when exposed to elevated temperatures and mechanical stress [20]. In contrast, carbon fiber exhibits stability; are highly inert and non-absorbent; and do not experience hydrolysis reactions [108,109].

**Figure 4:**
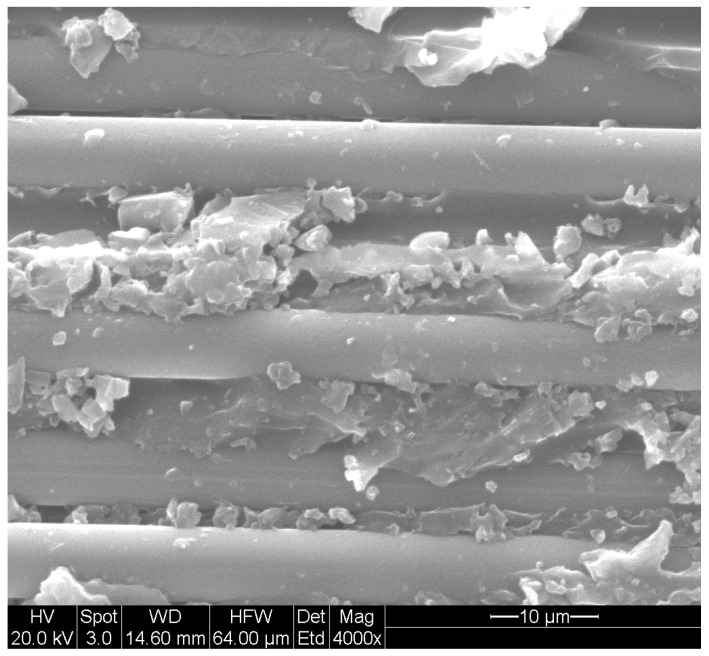
SEM of carbon/epoxy showing regions of fiber–matrix debonding.

The degradation of either the matrix or fiber can significantly weaken the interface, making it a region highly susceptible to deterioration and can lead to fiber–matrix debonding (Figure 4), which subsequently enables higher rates of moisture uptake through wicking. The interface in FRP serves as the boundary where fibers intersect with the polymer matrix, enabling load transfer based on strain compatibility. When this interface fails prematurely, the matrix’s deformation or strain no longer matches that of the fibers, preventing effective load transfer from the matrix (the weaker component) to the fibers (the stronger component), thus negating the reinforcing effect of the fibers on the polymer matrix. Therefore, maintaining strain compatibility at the interface is crucial for FRP material integrity. However, achieving this compatibility is challenging under conditions of stress or environmental exposure to moisture and humidity due to mechanical (e.g., elastic modulus) and physical property differences (e.g., swelling ratio) between fibers and the matrix [110].

Polymers commonly display a moisture uptake coefficient, denoted as *β*, which establishes a connection between the weight variation of the polymer due to the sorption of a solvent, like water, and corresponding volumetric alterations. This volume change is employed to calculate moisture-induced strain, using the moisture uptake values, where swelling remains negligible [43].

(2) $$\varepsilon =\beta \left(M_{t}-M_{0}\right)$$

where *ε* is the moisture-induced strain, *M_t_* is the moisture uptake value at time *t*, and *M*_0_ refers to the reference moisture uptake level when no swelling occurs. Water molecules disrupt polymer interchain bonding via hydrogen bonds and clusters, causing dimensional changes in the material. In the case of composite materials, the moisture uptake coefficient of glass fibers is significantly lower than that of typical polymer composite matrices, while other fibers, such as carbon, exhibit negligible moisture uptake [111]. Consequently, in unidirectional composites where fibers are in the longitudinal direction, minimal swelling is observed. However, noticeable dimensional changes occur in the transverse and through-thickness directions with increased moisture uptake [111]. The ingress of moisture induces a variable stress state before reaching saturation, and this stress distribution is contingent on the moisture concentration. According to Equation (2), delineating moisture-induced swelling strain, there exists a specific threshold moisture content that initiates a shift from compression to tension.

As water diffuses into the composite, stress gradients emerge at both macroscopic and interfacial levels. The layers with higher water concentration develop compressive moisture stresses, necessitating the development of tensile stresses further inward to maintain stress equilibrium within the bulk material [112]. These tensile stresses may induce microcracking, particularly in resin-rich areas surrounding voids [37]. Subsequently, these microcracks propagate through the resin toward interfaces, where the initiation of interfacial debonding may occur. Moisture-induced compressive stresses could contribute to the relaxation of residual tensile stresses established by thermal gradients during curing processes, potentially resulting in a strength increase before succumbing to moisture-induced resin and interfacial degradation [37]. The varying stress states occurring within the fiber–interphase–matrix region exert tensile forces on the fiber and compressive stresses on the matrix. Depending on the loading condition and material parameters [113], the interphase may experience either tensile or compressive stress, leading to significant internal stress gradients [113,114,115]. As the moisture content increases, the swelling strain progressively becomes more compressive. In unidirectional composites, the most substantial stresses emerge perpendicular to the reinforcing fiber. As moisture ingress takes place, the sum of forces through the thickness must equate to zero. Therefore, it is evident that the center of a sorbing species undergoes tensile loading, leading to microcracking, as polymers generally exhibit weakness in tension [116].

Additionally, differential swelling between the fiber and matrix can cause microcracks at their interface. For example, in carbon fiber polymer composites, the matrix absorbs moisture while carbon fibers do not, resulting in a mismatch in volumetric expansion. The swelling mismatch leads to microcracks during early exposure to moisture. Over longer-term exposure periods, these microcracks evolve into new paths for moisture transport, increasing morphological changes in the polymer and causing damage growth at the interface. Absorbed water can also generate osmotic pressure, potentially sufficient to cause debonding between the fiber and matrix along the interface, resulting in increased moisture sorption due to wicking [103,117,118].

Fiber–matrix debonding can lead to the potential dissolution of some matrix material into water and subsequent leaching along the interface due to hydrolysis [55,110,119,120,121]. Due to the interfacial debonding, the fiber reinforcement becomes vulnerable to hydrolytic attack. In carbon-fiber-reinforced polymers, the carbon fibers themselves are not affected by moisture or hydrolytic attack. However, the bond between the fibers and the matrix experiences a reduction in strength due to the decline in interfacial adhesion [122,123,124]. The combined effects of microcracking, high interfacial stress, and hydrolytic attack highlight the significant susceptibility of the interphasial region. As is discussed later, the presence of fibers causes a barrier to moisture transport with the passage being easier along the fiber than across it. This results in the diffusion coefficient along the length of fibers being higher than in the transverse direction [125,126]. A higher longitudinal diffusion coefficient is anticipated when interfacial debonding occurs, as water uptake through capillary action [116] resulting in an elevated rate of sorption along the interphase [67], where polymer characteristics may deviate from the bulk resin due to the resin’s interaction with the fiber and sizing [67,127].

The simultaneous occurrence of physical and chemical degradation processes may impact the polymer at the surface of the composite and weaken the strength of the polymer, potentially leading to surface erosion [123,128,129,130,131]. The formation of basic micro-voids may even precede polymer blistering before surface erosion occurs. In structural composites, blistering can manifest when cracks develop within the polymer, forming macro-voids where water accumulates. The occurrence of surface erosion and cracking exacerbates the vulnerability of the fiber reinforcement, amplifying the overall degradation process.

Hygrothermal degradation due to moisture sorption can cause an increase in the molecular weight between crosslinks and a concurrent decrease in crosslink density of the polymer. The decline in crosslink density suggests that chain scission takes place in the presence of water at elevated temperatures. Classical rubber elasticity principles explain that the average molecular weight between crosslinks denoted as *M_c_* (generally expressed in g/mol) can be effectively described as:

(3) $$M_{c}=\frac{3RT\rho }{E_{r\;p}^{\prime }}$$

where *E′_rp_* represents the rubbery modulus of the polymer, *R* denotes the gas constant (8.3143 J/mol K), *T* signifies the temperature in Kelvin, and *ρ* is the density of the polymer. This equation can be modified for use in composite materials as follows:

(4) $$M_{c}=\frac{3RT\rho }{E_{rp}^{\prime }\left(1-V_{f}^{n}\right)}$$

where *V_f_* is the fiber volume fraction, and the exponent, *n*, is 1/3 for fillers [132] and 1 for unidirectional composites, assuming the transverse direction is representative of the resin effect [101]. It is important to note that while the molecular weight calculations provide an acceptable order of magnitude [132], they should not be considered absolute values. Rather, these molecular weights should be understood as providing a qualitative indication of the changes occurring in the chemical structure of the composite matrix due to moisture sorption.

## 3. Moisture Uptake

Moisture uptake can occur when polymers and polymer composites come either directly or indirectly in contact with aqueous solutions or humidity. While the level of moisture content in a polymer or composite eventually reaches the maximum level when immersed in solution [55], this level (*M*_∞_) can fluctuate through exposure to environments with varying levels of relative humidity [133].

The thermodynamic differences between saturated states of water vapor and liquid water are significant. The saturated state of water vapor, where the vapor pressure (*e*) equals the saturation pressure (*e_s_*), differs thermodynamically from water. In this state, two independent phases of one molecule can coexist with equal chemical potential at equilibrium. It has been assumed that exposure to 100% relative humidity (RH) is comparable to immersion in water, to obtain isotherms for the material [36,133].

This leads to a consideration of Schroeder’s paradox [134], which describes the inconsistency and difference observed in fully saturated moisture content when the material is exposed to saturated vapor conditions compared to immersion in water at a specific temperature. Explanations for this paradox differ based on the material under consideration and include attribution of this behavior to capillary condensation in a presumed porous layer on the material surface [135], as an artifact of measurement techniques [136], or the existence of multiple solutions to a thermodynamic equilibrium equation [137].

Based on studies related to the penetration of rubber by water, it is hypothesized that a pressure gradient near the interface between water and rubber is responsible for the observed difference between exposure to the saturated vapor and water [57]. An early study on rubber treatment indicated that rubber submerged in a water solution absorbs more than when suspended in the saturated vapor above the solution. This is because the rubber seeks to achieve infinite dilution in water [41]. While a similar moisture absorption response has been observed in some polymer composites, showing reduced moisture absorption in saturated steam environments compared to water immersion at equivalent temperatures [138,139], others have been reported contrasting behavior. For example, Choi et al. [140] reported that carbon/epoxy systems can experience a greater moisture uptake in a 95% relative humidity environment than through immersion in water at the same temperature. Understanding these variations is crucial for assessing the sorption phenomena in polymer composites exposed to humid environments. It is emphasized that a single level of relative humidity can represent entirely different environments at different temperatures. In such cases, higher temperatures result in moist air containing a greater amount of water. Although water content in air is highly dependent on temperature, adsorption isotherms indicate that the relative pressure (or relative humidity for water vapor) determines the amount of vapor absorbed by a sorbent [80].

To better understand the role and effect of humidity on polymer/composite response it is important to have clarity on the phenomena itself. Relative humidity (RH) can be defined as the relationship between the actual water content in the air, indicated by its partial pressure *e*, and the maximum amount of water vapor the air can hold at the same temperature, known as saturation pressure *e_s_*, as follows:

(5) $$\%RH=\frac{e}{e_{s}(T)}\times 100\%$$

Alternatively, relative humidity can also be expressed in terms of the ratio between the actual water vapor dry mass mixing ratio, denoted as *w*, and the maximum (or saturation) mixing ratio, represented by *w_s_*, at the ambient temperature and pressure conditions as follows:

(6) $$\%RH=\frac{w}{w_{s}}\times 100\%$$

While relative humidity provides insights into atmospheric moisture levels, absolute humidity serves as a measure of the density of water vapor within a mixture of moist air and can be derived from fundamental principles [141]. The absolute humidity, denoted as *d_v_*, is defined as follows:

(7) $$d_{v}=\frac{m_{v}}{V}$$

where *m_v_* is the mass of water vapor within a volume *V*, *e* is the partial pressure of the water vapor, *n_v_* represents the moles of water vapor, *R* is the universal gas constant, and *M_v_* as the molecular weight of water. The ideal gas law implies the following:

(8) $$d_{v}=\frac{m_{v}}{n_{v}RT/e}=\frac{m_{v}e}{\frac{m_{v}}{M_{v}}RT}=\frac{eM_{v}}{RT}$$

leading to the following:

(9) $$d_{v}=\left(217\frac{g\cdot K}{m^{3}\cdot \mathrm{mbar}}\right)\frac{e}{T}$$

The constant 217 in this equation results from combining several constants (217 = *M_v_*/*R*), where *M_v_*≈ 18.02 g/mol and *R* ≈ 0.0831 L·bar/mol K). This equation shows how absolute humidity varies with temperature and relative humidity.

Isotherms are used to represent the maximum moisture content of a sorbent material as a function of partial pressure, defined as (*P/P*_0_), where *P* and *P*_0_ represent vapor pressure and saturation pressure, respectively, and represents how much moisture a substance can hold at equilibrium relative to its partial pressure [47,80]. With a basis in adsorption theory [47,80,142], isotherms, provide the theoretical basis for in the adsorption of molecules onto a surface. Incorporating thermal dependency into isotherm coefficients using isosteres [142] provides a means to introduce thermal dependency into the maximum moisture content and thus provides a means of assessing the effects of partial pressure (or humidity) and temperature on uptake.

**Figure 5:**
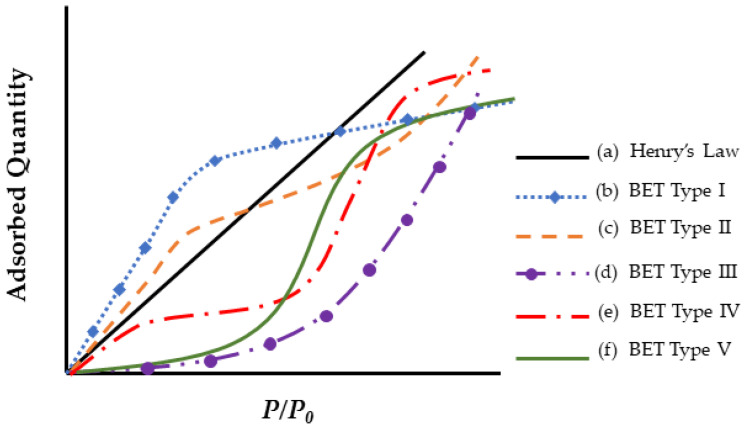
Schematic representation of several types of isotherms. (Partial pressure (*P*/*P*_0_) is shown in terms of *P* ≡ vapor pressure and *P*/*P*_0_ ≡ saturation pressure).

Brunauer–Emmett–Teller (BET) [143] isotherms are used to graphically represent the relationship between the amount absorbed on a material’s surface and the relative pressure at a constant temperature. Despite the challenge of obtaining comprehensive data across relative pressures, existing studies indicate that the equilibrium moisture content does not reach a plateau at high relative pressures. Instead, the isotherms may take a range of forms as depicted in Figure 5. Under significantly high and low pressures, the dual sorption isotherm simplifies to a linear structure, featuring two distinct slopes at high and low relative pressures, connected by a nonlinear region.

The sorption curve follows a linear pattern at low activities, with the quantity of absorbed water at equilibrium rising nearly linearly across a broad spectrum of water activities, following Henry’s law isotherm, except at low and high relative humidity. At low relative humidity, the deviation typically manifests as “type I” (curve (b) in Figure 5) isotherms, resembling Langmuir-type sorption isotherms (Brunauer–Emmett–Teller (BET) [143]. This type represents adsorption in a unimolecular layer. It is characterized by a rapid initial uptake of the adsorbate that quickly reaches a plateau, indicating the saturation of the adsorbent surface. Meanwhile, curve (c) in Figure 5 exhibits an S-shaped or sigmoid isotherm and is illustrative of a BET type II isotherm [143] under the dual sorption theory. The type II isotherm is the common type in BET analysis, showing a flatter region in the middle for monolayer formation, followed by multilayer formation at medium pressures. In curves (c) and (d) in Figure 5, adsorption increases as the vapor pressure of the adsorbed gas is approached, while in curves (e) and (f) in Figure 1, the maximum adsorption is attained, or almost attained, at some pressure lower than the vapor pressure of the gas [143]. Conversely, with high water activity, a deviation from this linearity occurs. The uptake of water at equilibrium increases markedly, potentially due to water clustering and the effect of temperature [144].

It is generally accepted that the amount of sorbate absorbed is directly proportional to the partial pressure, indicative of the amount of sorbate in the air. Henry’s law, in the context of equilibrium moisture content, explains this principle [145] as follows:

(10) $$M_{\infty }=k\;\left(\%RH\right)$$

where *M*_∞_ represents the equilibrium moisture content, (%RH) represents the percentage relative humidity level, and *k* is referred to as Henry’s law constant, emphasizing that higher relative humidity corresponds to greater equilibrium/saturation mass uptake (*M*_∞_) [139,146]. Henry’s law has limited applicability to composites with a modified expression. Freundlich’s equation can be expressedas:

(11) $$M_{\infty }=a{\left(\%RH\right)}^{b}$$

where the constants *a* and *b* are specific to the material type, derived through a linear regression analysis of the moisture content data [147], which has been shown to have better applicability [133]. The exponent *b* has been reported to have values ranging between 1 and 4.3 [36,82,139,146], although it should theoretically be less than 1 [148]. Typical values of exponents are given as examples in Table 2. As can be seen, the values of a are higher for the neat resin than for the composite. Furthermore, both exponents increase with an increase in temperature of exposure, indicating the dependence of the values on temperature.

Flory [149] and Huggins [150] developed a theory to describe polymer sorption, employing statistical analysis of polymer/solvent configurations using a lattice concept to model the random mixing of polymer and solvent, which was further developed by Apicella et al. [69]. The Flory–Huggins theory is frequently applied to correlate penetrant activity with solution composition, formulated as follows:

(12) $$\ln a_{s}=\ln\frac{P}{e}=\ln v+\left(1-v\right)+\chi {\left(1-v\right)}^{2}$$

where *a_s_* denotes solvent activity, which is directly related to relative humidity; *υ* indicates volume fraction of the solvent; *χ* signifies the interaction coefficient of polymer–solvent, which can be temperature dependent; and *P* is the equilibrium pressure, with *e* denoting partial pressure.

It is important that the role of these simple expressions (Henry’s law and Freundlich’s relation) is understood in the context of models developed for diffusion and hence a brief summary is provided herein. For example, the Langmuir model [151] is based on the following:

(13) $$M_{\infty }=\frac{c(\%RH)}{1+d(\%RH)}$$

whereas the dual sorption theory [144,152] addresses deviations from Henry’s law by combining Langmuir sorption [151] with Henry’s law [145], leading to the following:

(14) $$M_{\infty }=k(\%RH)+\frac{c(\%RH)}{1+d(\%RH)}$$

where *k*, *c*, and *d* can be expressed using Henry’s law dissolution constant, determined through the statistical thermodynamic analysis of Langmuir’s theory, such that

(15) $$k=k_{D}p_{0},\;\;\;c=C_{H}^{\prime }bp_{0},\;\mathrm{and}\;\;\;\;d=bp_{0}$$

where *k_D_* is the hole affinity constant, *b* is the hole saturation constant, *C_H′_* is the Langmuir capacity constant, and *p*_0_ is the saturation vapor pressure.

Essentially, the dual sorption theory operates on the premise that water initially permeates the sorbent as free water through normal diffusion processes, following Henry’s law, and can subsequently transform into an immobilized form as bound water at points within the micro-heterogeneous medium, characterized by its equilibrium conforming to the Langmuir isotherm [144]. When considering the kinetic aspect of dual sorption theory, known as Langmuir diffusion, additional assumptions come into play, which are further discussed in subsequent sections.

## 4. Moisture Uptake and Diffusion

Gravimetric measurement techniques are commonly used to record moisture uptake of fiber-reinforced polymer (FRP) composites [153]. Samples are immersed in solution at a constant temperature and the absorption of water is monitored over time during immersion, enabling the observation of sorbate diffusion into a single sorbent specimen over time and providing the overall mass change due to sorbate uptake or loss, rather than concentrating solely on concentration [154,155]. The determination of moisture content at any time *t*, *M_t_*, is specified as follows:

(16) $$M_{t}=\frac{w_{t}-w_{0}}{w_{0}}\times 100$$

where *w_t_* is the mass of the sample immersed in water at time *t*, and *w*_0_ refers to the initial mass of the specimen before immersion. In cases where there is no material degradation, water content increases with immersion time an initial segment where moisture content increases linearly with time, followed by the attainment of an asymptote known as the equilibrium water content. The duration of immersion time significantly affects the uptake profile. However, there are cases where the moisture uptake does not reach equilibrium through the exposure period and instead continues to increase as polymer chains rearrange over time due to moisture penetrant molecules, resulting in further absorption at a slower rate than the initial uptake. This prolonged exposure to moisture significantly affects the physical, chemical, and mechanical properties of the composites, highlighting the importance of understanding its impact for assessing their long-term behavior and durability in these applications [95,156].

**Figure 6:**
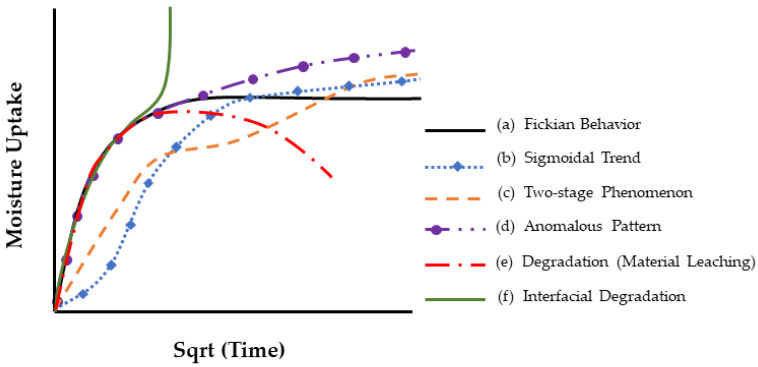
Characteristic moisture uptake profile in polymeric composites.

It is important to not just determine levels of uptake, but also the overall profile as represented by moisture content *M_t_* plotted against the square root of time *t*^1/2^, since these can provide rapid insight into the mechanisms and effects of uptake. A representative set of generic profile shapes is shown in Figure 6. These include the typical Fickian pattern, characterized by linear initial uptake up to around 0.6*M_t_/M*_∞_ and concave asymptotically towards the *x*-axis as *M_t_* approaches the system’s equilibrium content, *M*_∞_. Alternatively, non-Fickian sorption may present itself with initial sigmoidal uptake, two-stage phenomena, or other anomalous features. Sigmoidal sorption, illustrated in Figure 6, represents characteristic non-Fickian behavior. The curves exhibit an S-shaped pattern, indicating a point of inflection. In the early 1960s, Long and Richman [157] introduced the variable surface concentration (VCS) model, which provided a detailed interpretation of experimental observations and was likely the first to reasonably explain two-stage sorption behavior [158]. They proposed that while the transport process follows Fickian principles, the delayed attainment of equilibrium at the material surface leads to unusual kinetics.

Two-stage sorption depicts a commonly reported profile for moisture uptake wherein the uptake curve consists of two distinct segments: (1) rapid Fickian absorption and (2) slower non-Fickian absorption. The curve initially follows a Fickian pattern until it starts to plateau. Instead of reaching the saturation level typical for Fickian absorption, the curve extends into a non-Fickian region. Ultimately, saturation is achieved over a longer period of exposure. A theory providing a satisfactory explanation for the characteristics of two-stage sorption was proposed in 1978 by Berens and Hopfenberg [159]. Severe departures from the commonly assumed Fickian response are shown in Figure 2, marked (e) and (f), and are associated with weight loss and a rapid increase in fluid content, respectively. Sorption patterns initially showing the uptake of sorbate may eventually decline after reaching a maximum uptake, as illustrated by curve (e), suggesting sorbent loss. Curves exhibiting sudden uptakes after an apparent equilibrium, like the one in curve (f), signify sorbent breakdown, such as interfacial wicking in composites, resulting in significant increases in moisture content. Weight gains along curves b-d, suggest reversible or, nearly reversible, effects of fluids on polymeric composites, while weight gains along curves (e) and (f) indicate irreversible fluid-induced damage that may cause permanent material property degradation. Weight gain recordings along curve (f) are associated with extensive fiber–matrix debonding and degradation, while curve (e) indicates significant material loss due to the leaching of material (polymer or fiber), both exhibiting irreversible absorption behavior. It is emphasized that profiles obtained at one exposure condition could change dramatically through changes in exposure temperature, stress level, fluid acidity, or exposure duration [160,161].

Several foundational assumptions underlie the interpretation of moisture uptake curves. Primarily, it is presupposed that an increase in weight corresponds to an increase in the sorbate population within the sorbent. When the sorbate is extracted to retroactively calculate true weight gain post-degradation, the weight loss is considered to signify the removal of all sorbates and no sorbent. In other words, if LMWS are leached into the exposure environment, it is assumed that no additional LMWS are leached into the desorption environment. It is further assumed that impurities, such as metals or salts in solution, do not diffuse into the sorbent. Prior investigations indicate that solutions containing ions do indeed absorb into the specimen, but the modes of transport differ from those of water molecules [162].

Figure 5 and Figure 6 present two different depictions of isotherm graphs for fiber-reinforced polymer (FRP) composites, illustrating different aspects of moisture interaction with the material. Figure 5 shows adsorbed quantity versus relative pressure, representing adsorption on the material’s surface and how water vapor adheres as relative humidity increases. This graph typically displays relative pressure (*P*/*P*_0_) on the *x*-axis and the amount of gas adsorbed (in cm^3^/g or mol/g) on the *y*-axis, providing insights into the composite’s surface properties and porosity. Figure 6, on the other hand, depicts the bulk absorption of water into the material over time. The key differences between these isotherms include the physical processes they represent—surface adsorption in Figure 5 versus bulk absorption in Figure 6—their time dependency, and the information they provide. Adsorption isotherms, typically measured at equilibrium, offer insights into surface properties and porosity, while absorption isotherms, showing the kinetics of water uptake over time, inform about the material’s overall moisture uptake capacity and rate.

The process of water uptake in polymers can be analyzed through the lens of diffusion, which is the process by which matter moves from one part of a system to another due to random molecular motions. Heat transfer by conduction also occurs because of random molecular motions, and there is a clear similarity between these two processes. Fick [163] recognized this and was the first to quantify diffusion by using the mathematical equation for heat conduction, assuming steady-state flow. The mathematical theory of diffusion in isotropic substances is thus based on the idea that the rate at which a substance diffuses through a unit area of a section is proportional to the concentration gradient measured perpendicular to the section [49]. Adolf Fick’s first law [163] describes the diffusion process into a specific sorbing medium, as flux moving from regions of high concentration to those of low concentration as follows:

(17) $$J=-D\cdot \nabla C=-D\cdot \frac{dC}{dx}$$

where *J* represents the total one-dimensional flux and rate of transfer per unit area of section, and *D* stands for the diffusion coefficient. The diffusion coefficient is defined in units of area per time, illustrating the rate of diffusion into or out of a sorbent medium. The concentration gradient along the *x*-axis is ∇*C* = *dC*/*dx*, where *x* signifies distance through the sorbate, *C* represents the concentration of sorbate per unit of sorbent, and *t* represents time. The negative sign in the equation indicates that diffusion occurs in the direction of decreasing concentration.

Fick’s second law [49] of diffusion explains how the concentration profile changes over time during diffusion. It states that the rate of change of concentration over time is proportional to the second derivative of concentration with respect to position in three dimensions, *x*, *y*, and *z*, with the diffusion coefficient *D* serving as the proportionality constant as follows:

(18) $$\frac{dC}{dt}=D\left(\frac{d^{2}C}{dx^{2}}+\frac{d^{2}C}{dy^{2}}+\frac{d^{2}C}{dz^{2}}\right)$$

When diffusion is simplified to a single dimension, with a concentration gradient solely along the *x*-axis, then the equation reduces to the following:

(19) $$\frac{dC}{dt}=\nabla \cdot \left[D\left(\bar{\nabla }C\right)\right]=D\left(\frac{d^{2}C}{dx^{2}}\right)$$

Adolf Fick’s contribution to diffusion theory extends to recognizing diffusion as a dynamic molecular process and discerning the differences between a steady state and true equilibrium. Fick’s laws fundamentally necessitate a diffusion coefficient to express the rate of diffusion and commonly require achieving an equilibrium content, where the uptake rate gradually diminishes to zero as equilibrium is reached. Material characteristics, geometry, processing factors, and environmental exposures are factors that can influence and describe the parameters.

The influence of temperature on the diffusion coefficient, as widely recognized, is crucial in understanding moisture absorption in polymer composites. Studies have extensively explored how the rate of uptake, characterized in part by the diffusion coefficient varies with factors such as polymer type [100,102], crosslink density [164], degree of cure [165,166,167], filler type [82,168], and filler amount [45,169,170]. While the diffusion coefficient may vary over time [121,171], changes typically correlate with evolving concentration gradients, stress conditions, or reaction fronts. Increasing the temperature accelerates aging by modifying the diffusion rate, mechanism, and solubility [172,173,174]. Increased temperature leads to a higher diffusion rate due to increased molecular mobility and greater free volume [172]. According to the theory of free volume outlined in [175], available space between molecules increases as the temperature rises, starting from zero at absolute zero. When applied to viscoelastic polymers, the theory proposes that the viscosity and stiffness of the material depend on the amount of free volume within it [176]. Therefore, adding a diluent, such as water, to a polymer is expected to increase the overall free volume of the system, enhancing the movement of the polymer network [177].

It is noted that the temperature-dependent phenomenon [178] is frequently characterized through the Arrhenius relationship.

(20) $$D=D_{o}\exp\left[\frac{-E_{a}}{RT}\right]$$

where *D_o_* represents temperature-independent empirical constant, *E_a_* is the activation energy, *R* is the universal gas constant, and *T* represents temperature in degrees Kelvin. The activation energy, which represents the threshold that must be overcome to activate a mechanism/state, can be determined from the slope of a plot of *ln*(*D*) versus (1/*T*), and this is seen to vary not just with resin and type of composite, but also the thickness of the composite and the nuances of processing, especially as related to the degree of cure. Bonniau and Bunsell [139] reported activation energies of 47. 2 kJ/mol K and 43.9 kJ/mol K for E-glass/epoxy composites using diamine and dicyandiamide hardeners, respectively, wherein the former showed Fickian response and the latter with lower energy showed a two-stepped response. Karbhari [84] based on wet layup of carbon/epoxy reported activation energies of 71.02 kJ/mol K, 61.18 kJ/mol K, and 63.37 kJ/mol K, for the neat epoxy, one-layer composite, and two-layer composite, respectively, emphasizing that the decrease in activation energy for the composite was due to water-uptake-induced damage at the fiber–matrix interphase level.

However, the Arrhenius model may not always accurately describe the influence of temperature on degradation kinetics. Experiments have shown deviations from Arrhenius behavior, such as curvature instead of linear response, particularly in studies of polymer degradation [179], indicating that the overall lifetime of materials can be represented as the sum of individual processes, where the combined Arrhenius terms may exhibit non-Arrhenius behavior. Thus, while the Arrhenius relationship is useful for describing the thermal dependency of *D*, by determining *D_o_* and *E_a_*, a dual Arrhenius model [179] can be expressed as:

(21) $$D=D_{o1}\exp\left[\frac{-E_{a1}}{RT}\right]+D_{o2}\exp\left[\frac{-E_{a2}}{RT}\right]$$

where *D_o_*_1_ and *E_a_*_1_ represent the empirical diffusion coefficient and activation energy of the first phase, and *D_o_*_2_ and *E_a_*_2_ denote the corresponding values for the second phase.

While the Arrhenius model provides a reasonable approach for predicting the long-term behavior of FRP composites based on moisture uptake trends, it operates under the assumption that the dominant degradation mechanisms of the material remain constant over time and temperature during exposure, but the rate of degradation increases with the increase in temperature [180,181,182]. Zhou et al. [182], among others, presented an approach to calculate the time shift factor (TSF), between two solutions with the assumption that the Arrhenius time–concentration relationship remains applicable across the complete concentration range. Wu et al. [183] also recently employed a temperature shift factor (TSF) across various temperatures to forecast the prolonged effectiveness of Basalt fiber-reinforced polymer (BFRP) bars in an alkaline solution as follows:

(22) $$\mathrm{TSF}=\exp\left[\frac{E_{a}}{R}\left(\frac{1}{T_{0}}-\frac{1}{T_{1}}\right)\right]$$

where *T*_0_ and *T*_1_ denote temperatures pertaining to Arrhenius plots.

The activation energy (*E_a_*) for diffusion provides insight into the energy threshold needed for moisture diffusion. For instance, a lower activation energy of the resin suggests a weaker diffusion barrier and thus greater moisture absorption. The activation energy required for moisture diffusion depends on the type of water absorbed in the polymer. When water molecules enter a bulk polymer sorbent, they may exist in two states: free water and bound water [25,184]. Zhou and Lucas [62] investigated the mobility of water in different epoxy systems and reported that water molecules adhere to epoxy resins through hydrogen bonding, identifying two types of water in epoxy resins based on differences in the bond complex and activation energy. The quantity of bound water strongly depends on the exposure temperature, duration of exposure, and hygrothermal condition. Higher temperatures and longer exposure times result in a greater amount of bound water. Zhou and Lucas [25] observed a notable difference in activation energy required for desorbing bound water (around 15 kcal/mol) compared to free water (around 10 kcal/mol). This contrast in activation energies indicates variations in the binding strengths between water molecules and the resin and provides an explanation for deviation from the Fickian profile in Figure 2.

The understanding of effects of solution and humidity on moisture uptake in polymers highlights the importance of considering sample geometry in experiments. When measuring moisture absorption in materials, small plate-shaped samples are often used, because the time required for saturation increases with the square of the panel thickness. These samples are typically sized such that one dimension is much smaller than the other two, leading to moisture uptake mainly through the broad faces of the plate. The moisture concentration can then be approximated using the solution for diffusion in an infinite plate, resulting in a linear increase in total moisture content with the square root of time (*t*^1/2^) during the initial phase of absorption [133,170,185]. However, in practice, moisture uptake is not one dimensional. The width–thickness and length–thickness ratios of specimens, impact moisture absorption, resulting in the importance of accounting for edge effects. The edges of the composite samples are prone to localized moisture ingress due to surface flaws and exposed fibers known as the edge effect. Neglecting edge correction factors to account for this discrepancy can lead to inaccurate results and underestimate the overall moisture absorption characteristics of the composite material.

**Figure 7:**
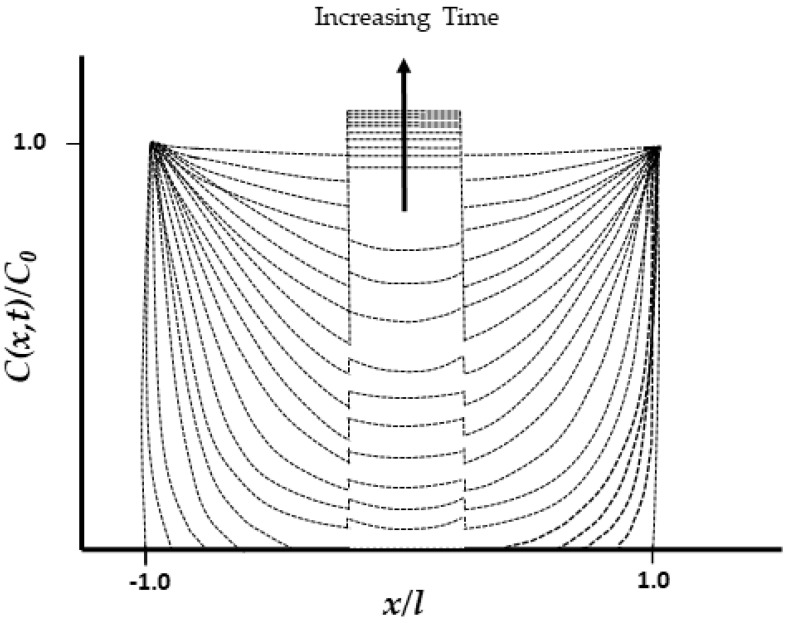
Moisture concentration distribution at various times along the thickness direction.

Figure 7, as an example, presents the distribution of moisture concentration *C*(*x*,*t*) normalized by the initial concentration *C*_0_ as a function of the position *x*/*l* in a symmetric, thin, unidirectionally reinforced composite sample of thickness 2*l*, where the thickness coordinate *x* ranges from −*l* to *l*, originating from the midplane. The curves are plotted for different time steps, with the direction of increasing time indicated by the arrow. Fluid absorption leads to material expansion, causing a non-uniform variation across the sample’s thickness during the transient diffusion state. This results in time-dependent, non-uniform in-plane stress, denoted as *C*(*x*,*t*). Higher moisture concentration exists near the edges until saturation is reached [186]. Furthermore, the presence of fibers at the edges facilitates a pathway for increased absorption, leading to more degradation in that area. The higher moisture uptake at the edges is accelerated by capillary action and interfacial wicking through microcracks and along debonded fiber–matrix interfaces, leading to a dramatic increase in moisture content. At saturation, the difference in moisture concentration across the specimen approaches zero, showing a uniform distribution. To compensate for water penetrating through the edges, the “edge effect” must be considered, and adjustments must be made for moisture uptake through all surfaces and edges [187]. It needs to be considered that while thin specimens absorb moisture faster, thick specimens take longer to reach saturation, making the use of edge correction factors more important.

## 5. Diffusion Models

The discussion in the previous section focused on mechanisms of uptake and basic theories. However, what is needed is full models that describe uptake as a function of time and consider the various factors that influence rate, level, and maximum moisture uptake.

Uptake is representative of absorption, which involves both solubility and diffusivity, underlining the essential understanding of moisture diffusion mechanisms in polymer matrix composites. Upon direct contact with the composite, liquid molecules penetrate the polymer surface and move through its bulk, driven by concentration gradients [38,188]. This process is characterized by the random motion and the rate at which liquid molecules move within the composite’s bulk. Polymers exhibit various diffusion behaviors, according to the relative rates of diffusion and polymer relaxation [49,51].

Selecting appropriate models for analyzing moisture uptake in fiber-reinforced polymer composites is important as the moisture uptake behavior often deviates from the Fick model as shown schematically in Figure 6.

The consequences of choosing an inappropriate model include premature termination of gravimetric monitoring of materials, failure to detect degradation phenomena that occur over longer absorption times, inaccurate estimates of maximum and saturation moisture uptake values, and misleading material characterization. Therefore, careful consideration in model selection is essential to accurately analyze moisture absorption behavior in polymer composites. Moreover, accurately determining diffusivity and moisture uptake within a polymer composite is crucial for predicting moisture-induced degradation effectively.

Among these models, one frequently utilized for steady-state one-dimensional diffusion is described by Fick’s laws [163], owing to its simplicity. Case I Fickian diffusion entails a diffusion rate unaffected by concentration. This occurs in polymers when the mobility of polymer segments is greater than that of the diffusing molecules, allowing liquid molecules to have minimal impact on the process. Rubbery polymers typically demonstrate Fickian diffusion due to their rapid response to environmental changes. Additionally, many glassy polymers also display Fickian diffusion under conditions where liquid solubility levels are relatively low. Case II Fickian diffusion, on the other hand, describes a situation where the diffusion rate is strongly influenced by concentration. This phenomenon is notable when absorbed liquid enhances polymer segment mobility via plasticization, thus easing diffusion. Case II Fickian diffusion commonly occurs when the diffusion rate exceeds polymer segment mobility, particularly observed in glassy polymers and with highly absorbent liquids. Non-Fickian or anomalous diffusion occurs when the mobility of polymer segments is comparable to the diffusion rate. This type of diffusion represents an intermediate state between the distinct behaviors observed in case I and case II diffusion.

The Fickian diffusion model offers a direct method to assess the water absorption of polymer composites. The conventional treatment of Fickian diffusion involves analyzing the one-dimensional aspect of Fick’s second law shown by Equation (19) [155,166,189,190,191,192,193,194]. Fick’s laws were developed based on fundamental heat-transfer equations, resulting in well-established solutions for the plane sheet case [49,195]. For an infinite plate, the moisture content in the isotropic (*D_x_ = D_y_ = D_z_ = D*) rectanguloid can be calculated assuming the materials follow 1-D Fickian behavior. The coefficient *D* is expected to remain constant regardless of time and position [196]. Considering a uniform initial distribution of sorbent, the solution to Fick’s second law can be expressed as follows [48,49]:

(23) $$\frac{C\left(x,t\right)-C_{0}}{C_{1}-C_{0}}=1-\frac{4}{\pi^{2}}\sum_{n=0}^{\infty }\frac{{\left(-1\right)}^{n}}{{\left(2n+1\right)}^{2}}\exp\left[-\frac{Dt}{h^{2}}\pi^{2}{\left(2n+1\right)}^{2}\right]\cos\left[\frac{\left(2n+1\right)\pi x}{h}\right]$$

where *D* is the diffusion coefficient, *C*_0_, is the initial distribution of sorbent within a plane sheet with surfaces held at a constant concentration, *C*_1_. This describes the moisture concentration distribution *C*(*x*,*t*) through the thickness of the plate, *x*, spanning from −*h*/2 to +*h*/2 for a plate thickness *h* over time, *t*. *M_t_*, the total moisture content over time, is determined by integrating *C*(*x*,*t*) through the thickness from −*h*/2 to +*h*/2 [122,189,197,198,199]:

(24) $$M_{t}=M_{\infty }\cdot \left\{1-\frac{8}{\pi^{2}}\sum_{n=0}^{\infty }\frac{1}{{\left(2n+1\right)}^{2}}\exp\left[-\frac{Dt}{h^{2}}\pi^{2}{\left(2n+1\right)}^{2}\right]\right\}$$

where *M*_∞_ is the equilibrium moisture uptake level.

It is fundamentally assumed that the mechanisms governing the diffusion of moisture into and out of a material are identical, leading to diffusion coefficients and trends for absorption and desorption. However, it has been consistently proven that this assumption should not be strictly followed because desorption trends fail to replicate absorption trends in many cases due to the presence of bound water [26,56,57,58,62,121].

**Figure 8:**
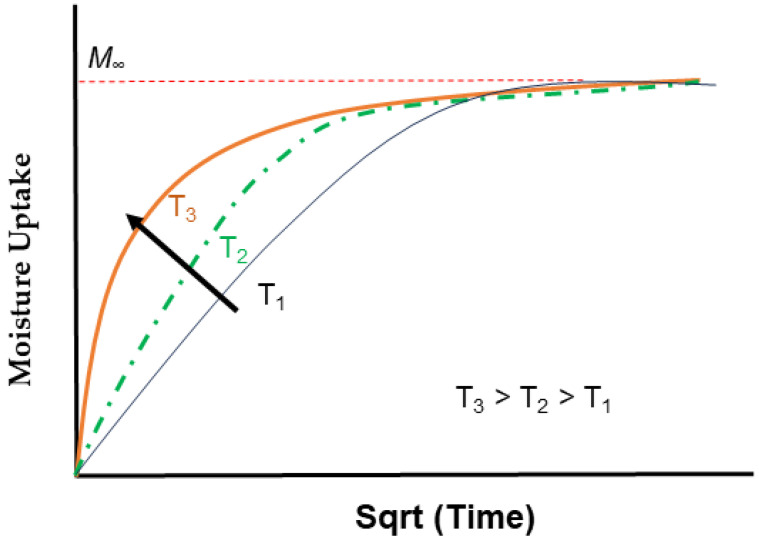
Schematic showing effect of temperature on rate and attainment of equilibrium for Fickian uptake.

Crank [49] proposed a short-time approximation for Equation (24) as follows:

(25) $$M_{t}=M_{\infty }\cdot \frac{4\sqrt{Dt}}{h}\cdot \left\{\frac{1}{\sqrt{\pi }}+2\sum_{n=1}^{\infty }{\left(-1\right)}^{n}\;\mathrm{ierfc}\;\frac{2\sqrt{Dt}}{nh}\right\}$$

which can be further simplified when *M_t_/M*_∞_ *<* 0.6 as suggested in [49,57,200,201] to the following:

(26) $$M_{t}=\frac{4M_{\infty }}{h}\cdot \sqrt{\frac{Dt}{\pi }}$$

emphasizing that the initial level of moisture absorption is directly proportional to the square root of time. This correlation is commonly used to determine if moisture uptake is Fickian [77,202,203,204], in addition to the requirement for attainment of an equilibrium level. Intrinsically, this suggests that an increase in temperature results in a higher rate of uptake and faster attainment of equilibrium as shown in Figure 8.

It should be noted that the use of Equation (26) is comparable to assuming that, during short times of uptake, a plane sheet can be regarded as a semi-infinite medium, exhibiting a uniform concentration within the sorbent, and a constant and consistently uniform concentration at the surface [49,57,133,195]. Grammatikos et al. [33] suggest that Equation (25) can be used to calculate moisture content over both long-term and short-term periods of exposure as follows:

(27) $$\frac{M_{t}}{M_{\infty }}=\frac{4}{\pi^{2}}\sqrt{\frac{Dt}{h^{2}}}\;\;\;\;\;\;\;\;\;when\;\;\;\;\;\;\;Dt/h^{2}<0.04$$

and

(28) $$\frac{M_{t}}{M_{\infty }}=1-\frac{8}{\pi^{2}}\exp\left(\frac{-Dt}{h^{2}}\pi^{2}\right)\;\;\;\;\;\;\;\;\;when\;\;\;\;Dt/h^{2}>0.04$$

**Figure 9:**
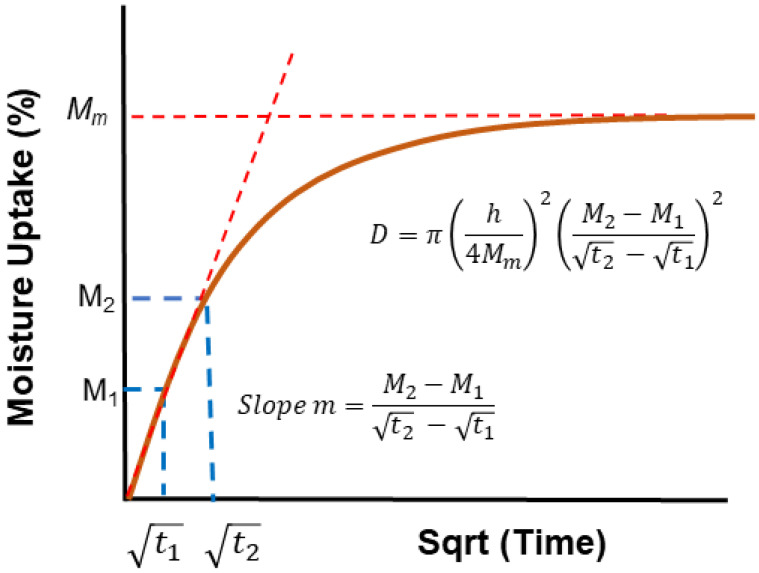
Schematic showing determination of diffusivity for Fickian uptake (following [133]).

Shen and Springer [133], based on a consideration of variation of the part of Equation (24) in parentheses with dimensionless time $t^{*}=\frac{D_{x}t}{s^{2}}$, suggested that:

(29) $$M_{t}\approx M_{\infty }\cdot \left\{1-\exp\left[-7.3{\left(\frac{Dt}{h^{2}}\right)}^{0.75}\right]\right\}$$

with diffusivity obtained from the initial slop of the uptake curve as shown in Figure 9.

Equation (29) is widely used for composites. The model, which is used extensively, overlooks the contribution of edge diffusion while providing reasonable estimates of *D* in the direction of fastest solution transport. However, edge diffusion becomes significant when the fastest diffusion direction aligns with the plate’s plane. Using a 1-D model can then potentially introduce significant errors, with discrepancies of up to 50% in derived diffusion coefficients [55]. Consequently, a 3-D Fickian model is often used with anisotropic diffusion coefficients following [204,205] as follows:

(30) $$\frac{M_{t}}{M_{\infty }}=1-{\left(\frac{8}{\pi^{2}}\right)}^{3}\sum_{i=0}^{\infty }\sum_{j=0}^{\infty }\sum_{k=0}^{\infty }\frac{\exp\left[-\pi^{2}t\left[D_{x}{\left(\frac{2i+1}{a}\right)}^{2}+D_{y}{\left(\frac{2j+1}{b}\right)}^{2}+D_{z}{\left(\frac{2k+1}{c}\right)}^{2}\right]\right]}{{\left(\left(2i+1\right)\left(2j+1\right)\left(2k+1\right)\right)}^{2}}$$

where *a*, *b*, and *c* represent the plate dimensions along the *x*-, *y*-, and *z*-axes, respectively, and *D_x_*, *D_y_*, and *D_z_* denote the diffusion coefficients along the corresponding axes. When experimentally determining diffusion coefficients, if the plate’s thickness is significantly smaller than its other dimensions, neglecting the edges is acceptable, and Equation (29) can be used to determine *D* values. Conversely, if edge effects on all six surfaces need to be considered, particularly when the sample’s thickness is comparable to its length and width, then edge correction factors, *f*, must be considered. For the case of anisotropic materials, Fick’s second law Equation (19), can be presented in Cartesian coordinates, incorporating distinct diffusion coefficients *D_x_*, *D_y_*, and *D_z_*, along the global *x*, *y*, and *z* directions, respectively [48]. In such cases, Equation (19) takes the following form:

(31) $$\frac{\partial C}{\partial t}=D_{x}\frac{\partial^{2}C}{\partial x^{2}}+D_{y}\frac{\partial^{2}C}{\partial y^{2}}+D_{z}\frac{\partial^{2}C}{\partial z^{2}}$$

Assuming that diffusion coefficients are independent of time and concentration, an effective diffusion coefficient can be introduced, analogous to approaches in heat transfer [195] as follows:

(32) $$\frac{\partial C}{\partial t}=D\left(\frac{\partial^{2}C}{\partial \varepsilon^{2}}+\frac{\partial^{2}C}{\partial \eta^{2}}+\frac{\partial^{2}C}{\partial \zeta^{2}}\right)$$

where *C* is the equilibrium concentration, and *ε*, *η*, and ζ represent the transformed coordinates defined as follows:

(33) $$\varepsilon =x\sqrt{\frac{D}{D_{x}}},\;\eta =y\sqrt{\frac{D}{D_{y}}}\;and\;\zeta =z\sqrt{\frac{D}{D_{z}}}$$

and *D_x_*, *D_y_*, and *D_z_* are diffusion coefficients along *x*, *y*, and *z* directions, respectively. In the case of non-steady-state diffusion into a thin plate with dimensions *l*, *w*, and *h* along the *x*, *y*, and *z* directions, respectively, where *h* is significantly smaller than *l* and *w*, a simplified relation between dimensions and diffusion coefficients was proposed by Jost [48]:

(34) $$\frac{l}{w}=\sqrt{\frac{D_{x}}{D_{y}}}$$

Given the fundamental assumption for diffusion into a thin plate of non-interference diffusion along the different directions, a supplementary derivation of the effective anisotropic diffusion coefficient, denoted as *D*, following the approach in [133] for a thin plate, leads to the following:

(35) $$\frac{\sqrt{D}}{h}=\frac{\sqrt{D_{x}}}{l}+\frac{\sqrt{D_{y}}}{w}+\frac{\sqrt{D_{z}}}{h}$$

where *D_x_*, *D_y_*, and *D_z_* rely on the orientation of the anisotropic medium under consideration, and *l*, *w*, and *h*, as before, are length, width, and thickness, respectively. In the case of unidirectional continuous fiber-reinforced composites, Shen and Springer [133] suggest the following:

(36) $$D_{a}=D_{x}{\left(1+\frac{h}{l}\sqrt{\frac{D_{z}}{D_{x}}}+\frac{h}{w}\sqrt{\frac{D_{y}}{D_{x}}}\right)}^{2}$$

where *D_x_*, *D_y_*, and *D_z_* represent the diffusion coefficients in the *x*, *y*, and *z* directions, respectively, with *z* designated as the fiber direction. Additionally, *h*, *w*, and *l* represent the specimen’s thickness, width, and length, respectively, with length corresponding to the fiber direction. Transverse diffusivities, in this case are typically considered equal i.e., *D_x_* = *D_y_*_,_ resulting in the following:

(37) $$\sqrt{D_{a}}=\left(1+\frac{h}{w}\right)\sqrt{D_{x}}+\frac{h}{l}\sqrt{D_{z}}$$

This method can be employed to ascertain the longitudinal and transverse diffusivities [16,206] by graphing √*Da* versus *h*/*l*. This plot typically yields a straight line, where the slope corresponds to *D_z_* and the intercept to (1 + (*h*/*w*)) √*D_x_*, where *D_x_* represents the adjusted 1-D diffusion coefficient, and *D_a_* represents the apparent coefficient determined through experimental data. In a homogenous material where *D* = *D_x_* = *D_y_*, Equation (35) can be expressed as follows:

(38) $$D=D_{x}{\left(1+\frac{h}{l}+\frac{h}{w}\right)}^{2}$$

In the most cases, the experimentally determined apparent diffusion coefficients use a one-dimensional approximation, neglecting additional diffusion through the edges. Various approximations have been proposed to alleviate this complexity in determining coefficients for true 3-D flow. Building on the approach of Shen and Springer [133], the studies by Bao and Yee [207] suggest that, in the early stage, the diffusion in the *x*, *y*, and *z* directions could be treated independently, assuming that the total mass of absorbed moisture equals the cumulative amount absorbed from each surface independently. Consequently, the moisture uptake can be expressed [192,208] as follows:

(39) $$\frac{M_{t}}{M_{\infty }}=\sqrt{\frac{16t}{\pi }}\left(\frac{\sqrt{D_{x}}}{l}+\frac{\sqrt{D_{y}}}{w}+\frac{\sqrt{D_{z}}}{h}\right)$$

While effective, this approximation overlooks diffusion at the sample edges. Therefore, Starink et al. [187] proposed an improved approximation, further discussed in [209,210,211,212]:

(40) $$\frac{M_{t}}{M_{\infty }}=\sqrt{\frac{16t}{\pi }}\left(\frac{\sqrt{D_{x}}}{l}+\frac{0.54\sqrt{D_{y}}}{w}+\frac{0.54\sqrt{D_{z}}}{h}+\frac{0.33l}{wh}\sqrt{\frac{D_{y}D_{z}}{D_{x}}}\right)$$

It should be noted that in an orthorhombic system, such as the case with unidirectional composite laminae, there exist three principal diffusivities—*D*_1_, *D*_2_, and *D*_3_, where *D*_1_ aligns with the fiber axis, *D*_2_ extends transversely across the width of the composite (longitudinal and transverse directions), and *D*_3_ represents the through-thickness diffusivity. Following the methodology of Jaeger and Carslaw [195], determination of principal diffusivities involves a translation from global *x*-*y*-*z* coordinates to local 1–2–3 coordinates, considering a fiber orientation described by angles *α*, *β*, and *γ* between the 1 axis and the *x*, *y*, and *z*-axes, respectively. In the context of laminar composites, where through-thickness properties closely resemble transverse properties across the width, it is reasonable to assume *D*_2_ *= D*_3_ [133,195], such that

(41) $$D_{x}=D_{1}\cos^{2}\alpha +D_{2}\sin^{2}\alpha$$

(42) $$D_{y}=D_{1}\cos^{2}\beta +D_{2}\sin^{2}\beta$$

(43) $$D_{z}=D_{1}\cos^{2}\gamma +D_{2}\sin^{2}\gamma$$

For unidirectional laminated composites, where *D*_1_ and *D*_2_ denote the diffusivities parallel and perpendicular to the fibers, respectively, and where *D_x_* is not known, diffusivity can be determined using the diffusivity of the bulk resin, *D_r_*_,_ and the fiber volume fraction, *V_f_*, as follows:

(44) $$D_{x}=D_{r}\left[\left(1-v_{f}\right)\cos^{2}\alpha +\left(1-2\sqrt{v_{f}/\pi }\right)\sin^{2}\alpha \right]$$

The diffusivity in the composite parallel to the direction of the fibers, *D*_1_, can then be determined by the rule of mixtures where the diffusivities of the resin and fibers within a composite are *D_r_* and *D_f_*, respectively:

(45) $$D_{1}=\left(1-V_{f}\right)D_{r}+V_{f}D_{f}$$

Further, following [213], Shen and Springer [133] develop an analogy for diffusivity as follows:

(46) $$D_{2}=\left(1-2\sqrt{\frac{V_{f}}{\pi }}\right)D_{r}+\frac{D_{r}}{B_{D}}\left[\pi -\frac{4}{\sqrt{1-B_{D}^{2}V_{f}/\pi }}\tan^{-1}\left(\frac{\sqrt{1-B_{D}^{2}V_{f}/\pi }}{1+\sqrt{B_{D}^{2}V_{f}/\pi }}\right)\right]$$

where

(47) $$B_{D}=2\left(\frac{D_{r}}{D_{f}}-1\right)$$

For composites in which the fiber’s diffusivity is lower than that of the resin, such as in the case of as carbon and basalt fibers, Equations (45) and (46) can be simplified for *V_f_* < 0.785 as follows:

(48) $$D_{1}≅\left(1-V_{f}\right)D_{r}$$

(49) $$D_{2}≅\left(1-2\sqrt{\frac{V_{f}}{\pi }}\right)D_{r}$$

where *D*_1_ and *D*_2_ denote the diffusivities parallel and perpendicular to the fibers, respectively, and *D_r_* is the diffusivity of the bulk resin. The value 0.785 originates from the packing efficiency of a hexagonally close-packed structure, in which the maximum possible volume fraction that spheres (or fibers in composite materials) can occupy within a given space is denoted. Consequently, the integration of Equation (35) results in the following:

(50) $$\frac{\sqrt{D}}{h\sqrt{D_{r}}}=\frac{\sqrt{1-V_{f}\cos^{2}\alpha +2\sqrt{\frac{V_{f}}{\pi }}\sin^{2}\alpha }}{l}+\frac{\sqrt{1-V_{f}\cos^{2}\beta +2\sqrt{\frac{V_{f}}{\pi }}\sin^{2}\beta }}{w}+\frac{\sqrt{1-V_{f}\cos^{2}\gamma +2\sqrt{\frac{V_{f}}{\pi }}\sin^{2}\gamma }}{h}$$

or

(51) $$D=D_{r}\left[(1-V_{f})\cos^{2}\alpha +(1-2\sqrt{\frac{V_{f}}{\pi }}{)\sin}^{2}\alpha \right]\times {\left[1+\frac{h}{l}\sqrt{\frac{\left(1-V_{f}\right)\cos^{2}\beta +(1-2\sqrt{\frac{V_{f}}{\pi }}{)\sin}^{2}\beta }{(1-V_{f})\cos^{2}\alpha +(1-2\sqrt{\frac{V_{f}}{\pi }}{)\sin}^{2}\alpha }}+\frac{h}{w}\sqrt{\frac{\left(1-V_{f}\right)\cos^{2}\gamma +(1-2\sqrt{\frac{V_{f}}{\pi }}{)\sin}^{2}\gamma }{(1-V_{f})\cos^{2}\alpha +(1-2\sqrt{\frac{V_{f}}{\pi }}{)\sin}^{2}\alpha }}\right]}^{2}$$

where *α*, *β*, and *γ* represent the angles between the fiber direction and the *x*, *y*, and *z*-axes, respectively, and *h*, *l*, and *w* indicate the thickness, length, and width of the specimen. In the case of a unidirectional composite represented by Equation (50) with a fiber volume exceeding *V_f_* > 0.785, a simplified approximation can be expressed as follows:

(52) $$D≅D_{r}h^{2}{\left[\frac{\sqrt{1-2\sqrt{V_{f}/\pi }}}{h}+\frac{\sqrt{1-V_{f}}}{l}+\frac{\sqrt{1-2\sqrt{V_{f}/\pi }}}{w}\right]}^{2}$$

Although these statements theoretically hold true for *V_f_* > 0.785, it is important to note that practical *V_f_* values generally fall below 0.785. Theoretically calculated fiber volume fractions range from 78.5% for square packing of circular fibers to 90.7% for hexagonal packing of circular fibers [100]. Shen and Springer [133] and Starink et al. [187] derived correction factors for diffusivity coefficients based on dimensions and flow directions and these are given below:

(53) $$f_{S\&S}=1+\frac{a}{b}+\frac{a}{c}$$

(54) $$f_{SSC}=1+\lambda_{1}\frac{a}{b}+\lambda_{1}\frac{a}{c}+\lambda_{2}\frac{a^{2}}{bc}$$

where *a*, *b*, *c* are the dimensions of a rectangular solid (*a* ≤ *b* ≤ *c*), and *λ*_1_ and *λ*_2_ are the average moisture concentration based on the direction of exposure. Several methods exist to derive *λ*_1_ and *λ*_2_, with the most accurate obtained by fitting their values using the complete 3-D diffusion Equation (30) and calculating the slope of the initial part across a range of rectanguloid sample shapes, as recommended by Starink et al. [187] such that

(55) $$f_{SSC}=1+0.54\frac{a}{b}+0.54\frac{a}{c}+0.33\frac{a^{2}}{bc}$$

It should be noted that both correction factors assume that the average concentration is a constant, irrespective of specimen size. Based on a study of rectangular specimens of different sizes, Starink et al. [187] reported that Equation (55) is more accurate than Equation (53) with deviations in the diffusion coefficient being between 16 and 37% for the latter compared to less than 2% for the former.

In unidirectional composites, the diffusion rates can, in general, be expected to be direction dependent, and can be elucidated in three different cases:

Case 1: *D_f_* = 0 and *α* = 0

(56) $$D_{eff}\left(\alpha =0\right)\;≃D_{r}{\left[1+\lambda_{1}\left(\frac{a}{b}+\frac{a}{c}\right)\sqrt{\frac{1-2\sqrt{v_{f}/\pi }}{1-v_{f}}}\right]}^{2}$$

Case 2: *D_f_* = 0 and *β* = 0

(57) $$D_{eff}\left(\beta =0\right)≅D_{r}\frac{1-2\sqrt{v_{f}/\pi }}{1-v_{f}}\times {\left[1+\lambda_{1}\left(\frac{a}{b}\sqrt{\frac{1-v_{f}}{1-2\sqrt{v_{f}/\pi }}+\frac{a}{c}}\right)\right]}^{2}$$

Case 3: *D_f_* = 0 and *γ* = 0

(58) $$D_{\mathrm{eff}\;}\left(\gamma =0\right)≅D_{r}\frac{1-2\sqrt{v_{f}/\pi }}{1-v_{f}}\;\times {\left[1+\lambda_{1}\left(\frac{a}{b}+\frac{a}{c}\sqrt{\frac{1-v_{f}}{1-2\sqrt{v_{f}/\pi }}}\right)\right]}^{2}$$

Although the Fickian model is used extensively for polymers and fiber-reinforced polymer matrix composites, it must be noted that the model does not consider swelling and assumes that the rate of uptake/diffusion is significantly faster than that of relaxation and thus is unable to address effects of volumetric change induced by moisture uptake, nor those of stresses. Furthermore, there is also an implicit assumption that the equilibrium moisture content is insensitive to temperature. While the initial stages of water diffusion in polymer and polymer matrix composites typically follow Fickian behavior, there is a significant body of literature showing deviation over longer periods of exposure [49,110,120,121,124,132,139,171,184,199,200,204,214,215,216,217,218,219,220,221,222,223,224,225,226,227,228]. Anomalous diffusion, which occurs when deviations from Fickian behavior occur, is influenced by factors such as material type, environmental conditions, and the material’s exposure history and level of degradation [49,119]. It should be remembered that the movement and ingress of water molecules into a polymer depends not only on water sorption into the free volume but also on the complex interactions between the polymer network and water. Factors such as polar groups within the structure, crosslink density, available free volume, and the outcomes of water–polymer interactions (including segmental relaxation, swelling, plasticization, and structural degradation) result in complexities and competing phenomena, which cause deviation from the ideal response represented by Fick’s laws [66,229,230].

Mubashar et al. [231], Karbhari and Xian [16], and Jiang et al. [232] proposed that under low temperatures and when materials are exposed to humid air, the diffusion process typically follows a Fickian pattern. However, at higher temperatures and when materials are immersed in solutions, the diffusion process often diverges from Fickian behavior due to relaxation of the glassy polymer network and filling of debonded zones and voids through wicking [16,232]. As temperature or humidity increases, the predominance of Fickian diffusion mechanisms decreases, allowing other mechanisms to have a greater impact on moisture transport.

It should be remembered that moisture absorption in composite materials is through three different mechanisms: (1) diffusion of water molecules within the micro-gaps between polymer chains; (2) capillary transport into the gaps and defects at the interfaces between fibers and matrix; and (3) transport through microcracks within the matrix due to swelling [233]. The presence of polar functional groups within cured epoxy resins, along with the relaxation processes induced by interactions with water molecules, leads to a deviation from classical Fick’s law over time. Thus, the detailed understanding of diffusion mechanisms is important for modeling of uptake. In glassy polymers, the diffusion of small molecules often deviates from simple Fickian diffusion, as noted in numerous studies [49,227,228]. Consequently, several models have been introduced to address anomalous diffusion in resin [231,234,235,236,237]: two phase diffusion model (Jacobs–Jones model) [154,184,203,218,219,228,235,238,239,240,241,242], coupled diffusion–relaxation model (Berens–Hopfenberg model) [159,234], time-varying diffusion coefficient model [171,236,243], and the Langmuir-type model (Carter–Kibler model) [184,220,231], which was extended to the hindered diffusion model (HDM) [125,171,244,245], structural modification diffusion model [207], the stress-dependent model [49,227,228], and the history-dependent theory [49,228], thickness-dependent moisture absorption model [246], and barrier models [247,248]. These models incorporate additional factors, such as bound and unbound/free water phases, relaxation effects from swelling, various polymer-formed phase structures, and changes in diffusion coefficients over time. Empirical and experimental observations are used to determine their parameters and coefficients. It is widely accepted that transport in glassy polymers involves both concentration-gradient-driven Fickian diffusion and time-dependent relaxation processes [49,159,227,228,249]. Depending on the relative contributions of these two processes, a diverse range of behaviors has been observed [228].

As a means of addressing the discrepancy, Jacobs and Jones developed a model based on an assumption of simultaneous sorption initiation by two distinct Fickian phases [238]. One phase is denser, characterized by high crosslinking, while the other is less dense, with low crosslinking, thus indicating moisture uptake through two separate and independent diffusion phenomena, represented as follows:

(59) $$\frac{M_{t}}{M_{\infty }}=V_{d}\cdot \left\{1-\exp\left[-7.3{\left(\frac{D_{d}t}{h^{2}}\right)}^{0.75}\right]\right\}+\left(1-V_{d}\right)\cdot \left\{1-\exp\left[-7.3{\left(\frac{D_{l}t}{h^{2}}\right)}^{0.75}\right]\right\}$$

where *M_t_* represents the moisture uptake at time *t*, *M*_∞_ is the moisture content at saturation, *V_d_* is the volume fraction of the denser phase, *D_d_* is the diffusion coefficient of the denser phase, and *D_l_* is the diffusion coefficient of the less dense phase. The initial phase of water uptake is characterized by rapid uptake, and the rate of uptake is expected to be additive, where diffusion occurs in both the highly crosslinked and lower crosslinked phases. Subsequently, the diffusion process decelerates as water is absorbed primarily into the highly crosslinked phase, while the less dense phase is assumed to be saturated and approaches equilibrium [154,238]. Maggana and Pissis [154] generalized the concept of denser and less dense phases into two arbitrary and independent phases, wherein the primary phase (referred to as phase Ι), that is, homogeneous and nonpolar, is responsible for the majority of water absorption, alongside a secondary phase (referred to as phase ΙΙ), characterized by varying density and/or hydrophilic properties compared to phase Ι. Assuming that water diffusion occurs independently within each phase according to Fick’s second law, the diffusion coefficient *D* and the saturation water content *M* for each phase can be determined separately [154], leading to a modification of Equation (59) as follows:

(60) $$M_{t}=M_{1}\cdot \left\{1-\frac{8}{\pi^{2}}\sum_{n=0}^{\infty }\frac{1}{{\left(2n+1\right)}^{2}}\exp\left[-\frac{D_{1}t}{h^{2}}\pi^{2}{\left(2n+1\right)}^{2}\right]\right\}+M_{2}\cdot \left\{1-\frac{8}{\pi^{2}}\sum_{n=0}^{\infty }\frac{1}{{\left(2n+1\right)}^{2}}\exp\left[-\frac{D_{2}t}{h^{2}}\pi^{2}{\left(2n+1\right)}^{2}\right]\right\}$$

where *M*_1_ and *M*_2_ denote the maximum moisture content for phases Ι and ΙΙ, respectively; *D*_1_ and *D*_2_ are the diffusion coefficients for phases Ι and ΙΙ, respectively; *h* represents the thickness; and *t* is the time. Since the uptakes are additive,

(61) $$M_{\infty }=M_{1}+M_{2}$$

where *M*_∞_ is the total moisture content at saturation of two stages, while the diffusion coefficient of each stage follows the Arrhenius relationship with independent values for the factor *D_o_* and activation energy E_a_. Equation (60) can be modified following the approach of Shen and Springer [133]:

(62) $$M_{t}=M_{1}\cdot \left\{1-\exp\left[-7.3{\left(\frac{D_{1}t}{h^{2}}\right)}^{0.75}\right]\right\}+M_{2}\cdot \left\{1-\exp\left[-7.3{\left(\frac{D_{2}t}{h^{2}}\right)}^{0.75}\right]\right\}$$

**Figure 10:**
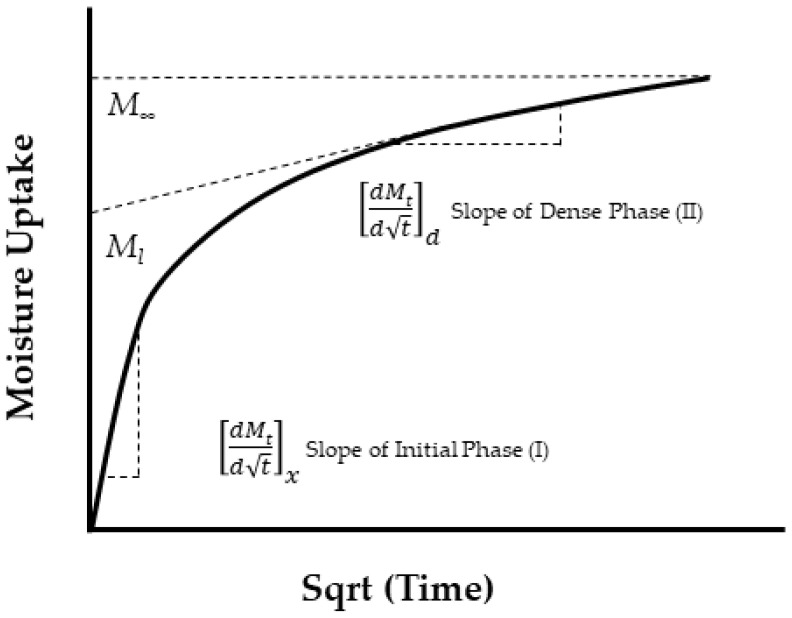
Schematic showing determination of parameters in the two-phase Fickian model. (*x* = *l* + *d*, *l* represents the less dense phase and *d* represents denser phase).

It is emphasized that the existence of two stages can follow directly from the existence of two distinct water phases within a resin subjected to hygrothermal conditions. The sorption of phase I is influenced by the atmospheric water content, while the sorption of phase II is governed by temperature and time [62]. Mikols et al., for example [21], propose a simplified model that considers water sorption in the free volume, characterized by maximum moisture content, and accounts for additional water sorption resulting from changes in the polymer structure due to hygrothermal interactions and changes in free volume [21]. The process of determining diffusion coefficients for Equation (59) involves employing linear curve fitting of regions predominantly governed by each phase. As shown in Figure 10, the initial slope of the uptake curve provides an approximate value for the diffusion coefficient, *D_x_*, following the conventional Fickian approach [238] such that

(63) $${\left[\frac{\partial M_{t}}{\partial \sqrt{t}}\right]}_{x}=\frac{4M_{\infty }}{h}\cdot \sqrt{\frac{D_{x}}{\pi }}$$

Since that uptake is additive as described by Equation (61) [238]:

(64) $${\left[\frac{\partial M_{t}}{\partial \sqrt{t}}\right]}_{x}={\left[\frac{\partial M_{t}}{\partial \sqrt{t}}\right]}_{d}+{\left[\frac{\partial M_{t}}{\partial \sqrt{t}}\right]}_{l}$$

where the subscripts *d* and *l* represent the denser and less dense phases, respectively, while *x* is the combination of *l* and *d*.

At later stages, the slope indicates the uptake in the denser phase, while the uptake in the less dense phase approaches equilibrium. By extending a line from the region characterized by sorption of the denser phase to the ordinate axis, the equilibrium content for the less dense phase, denoted as *M_l_*, can be determined from the intercept [238], as depicted in Figure 10. Subsequently

(65) $$M_{\infty }=M_{l}+M_{d}$$

enables the determination of the diffusion coefficients associated with it [238]:

(66) $${\left[\frac{\partial M_{t}}{\partial \sqrt{t}}\right]}_{d}=\frac{4M_{d}}{h}\cdot \sqrt{\frac{D_{d}}{\pi }}$$

and

(67) $${\left[\frac{\partial M_{t}}{\partial \sqrt{t}}\right]}_{l}=\frac{4M_{l}}{h}\cdot \sqrt{\frac{D_{l}}{\pi }}$$

such that,

(68) $$D_{d}=\pi {\left(\frac{h}{4\left[M_{\infty }-M_{l}\right]}\right)}^{2}{\left[\frac{\partial M_{t}}{\partial \sqrt{t}}\right]}_{d}^{2}$$

and

(69) $$D_{l}=\pi {\left(\frac{h}{4M_{l}}\right)}^{2}{\left\{{\left[\frac{\partial M_{t}}{\partial \sqrt{t}}\right]}_{x}-{\left[\frac{\partial M_{t}}{\partial \sqrt{t}}\right]}_{d}\right\}}^{2}$$

From Equations (59) and (60), it can be deduced that the volume fraction of both the more and less dense phases can be expressed as follows:

(70) $$V_{d}=\frac{M_{d}}{M_{\infty }}$$

and

(71) $$1-V_{d}=\frac{M_{l}}{M_{\infty }}$$

Jacobs and Jones [238] introduced a correlation for *V_d_* derived from a universal formula for thermal conductivity in two-component systems exhibiting orthorhombic symmetry, where

(72) $$V_{d}=\frac{\left(\frac{D_{d}}{D_{l}}+2\right)\left(\frac{D_{x}}{D_{l}}-1\right)}{\left(\frac{D_{d}}{D_{l}}-1\right)\left(\frac{D_{x}}{D_{l}}+2\right)}$$

where *V_d_* represents the volume fraction of the dense phase relative to the total system (highly crosslinked), while *D_d_* and *D_l_* denote the diffusion coefficients associated with the denser and less dense phases, respectively, and *D_x_* is the diffusion coefficient of the first stage of uptake.

In considering the influence of temperature on equilibrium moisture content, various approaches can be considered. One possibility involves a material capable of sorbing a finite amount of sorbate, where the volume percentage of one phase increases with temperature, resembling the model proposed in [21]. This implies that *M*_∞_ remains constant over temperature while *V_d_* varies. On the other hand, one could posit that there is no alteration in the relative volume percentage of either phase, but the equilibrium content of each phase increases proportionally with temperature. This suggests that *M*_∞_ varies with temperature while *V_d_* stays constant. A consideration in this vein is that of the levels of free and bound water within the polymer or composite as a function of period of exposure and details of the environmental conditions.

The Langmuir model and its variations consider the chemical interactions between water molecules and the polar groups of the resin, through assumption of the presence of two states for absorbed water molecules (expressed as two separate probabilities of state) and presents a framework where water sorption involves both the diffusion of free species into the sorbent and the simultaneous adsorption of water molecules to the polymer, forming a bound structure [144,184]. Carter and Kibler [184] proposed a non-steady-state diffusion model that integrated diffusion and Langmuir adsorption theory [46,144,203,231] such that

(73) $$\frac{dC_{D}}{dt}=-\frac{dq}{dx}-\sigma$$

where *x* represents position, *t* signifies time, *C_D_*(*x*,*t*) denotes the concentration of the free diffusion phase, *q*(*x*,*t*) indicates the mass flux of the free phase, and *σ*(*x*,*t*) represents the rate at which the free phase transitions into a site-bound state. It is crucial to emphasize that only the free phase undergoes diffusion, while both free and bound water molecules possess the capability to bind to and free themselves from sorbent molecules. By introducing the Langmuir diffusion coefficient, *D*, along with the parameters *α* and *β*, and considering the concentration of site-bound molecules, *C_S_*(*x*,*t*), the ensuing fundamental equations can be formulated as follows [184]:

(74) $$q=-D\frac{dC_{D}}{dx}$$

(75) $$\sigma =\beta C_{D}-\alpha C_{S}$$

where α signifies the probability of a bound molecule transitioning to a free state, *β* represents the probability of a free molecule becoming bound, *C_D_* is the concentration of the free diffusion phase, *C_s_* is the concentration of bound molecules, and *σ* represents the rate at which the free phase transitions into a bound phase. Therefore,

(76) $$\frac{\partial C_{D}}{\partial t}=D\frac{\partial^{2}C_{D}}{\partial x^{2}}-\beta C_{D}+\alpha C_{S}$$

At equilibrium, the conversion rate is indicated as follows [144,184]:

(77) $$\sigma =0,\;\;\beta C_{D}=\alpha C_{S}$$

and the maximum moisture content, *M*_∞_, at equilibrium can be described in terms of the equilibrium concentration *C_D_*_∞_ and *C_S_*_∞_ as follows:

(78) $$M_{\infty }=C_{D\infty }+C_{S\infty }=C_{D\infty }+\frac{\beta }{\alpha }C_{D\infty }$$

where *C_D_*_∞_ is the equilibrium concentration of the free diffusion phase, and *C_S_*_∞_ is the equilibrium concentration of bound molecules. Subsequently, the expression for moisture content *M_t_*, can be formulated as follows [184,203]:

(79) $$M_{t}=M_{\infty }\cdot \left\{1-\frac{\beta }{\alpha +\beta }\exp\left(-\alpha t\right)-\frac{\alpha }{\alpha +\beta }\exp\left(-\beta t\right)\cdot \frac{8}{\pi^{2}}\sum_{n=0}^{\infty }\frac{1}{{\left(2n+1\right)}^{2}}\exp\left[-\frac{Dt}{h^{2}}\pi^{2}{\left(2n+1\right)}^{2}\right]\right\}$$

where *t* represents time in seconds, *D* stands for the Langmuirian diffusion coefficient, and *h* denotes the thickness of the specimen. When both *α* and *β*, representing the probability of a bound molecule transitioning to a free state and a free molecule becoming bound, respectively, are small compared to the parameter governing the rate of saturation of a one-dimensional specimen with thickness *h*, Carter and Kibler introduced the term *κ* [184], such that

(80) $$\kappa =D{\left(\frac{\pi }{h}\right)}^{2}$$

and indicated that Equation (79) is applicable only when *α*, *β* << *κ*. Since the conversion rate between the two phases is expected to be significantly less than *κ*, Equation (79) can be restated without the exp(−*βt*) term as follows [38,139,203,250]:

(81) $$M_{t}=M_{\infty }\cdot \left\{1-\frac{\beta }{\alpha +\beta }\exp\left(-\alpha t\right)-\frac{\alpha }{\alpha +\beta }\cdot \frac{8}{\pi^{2}}\sum_{n=0}^{\infty }\frac{1}{{\left(2n+1\right)}^{2}}\exp\left[-\frac{Dt}{h^{2}}\pi^{2}{\left(2n+1\right)}^{2}\right]\right\}$$

**Figure 11:**
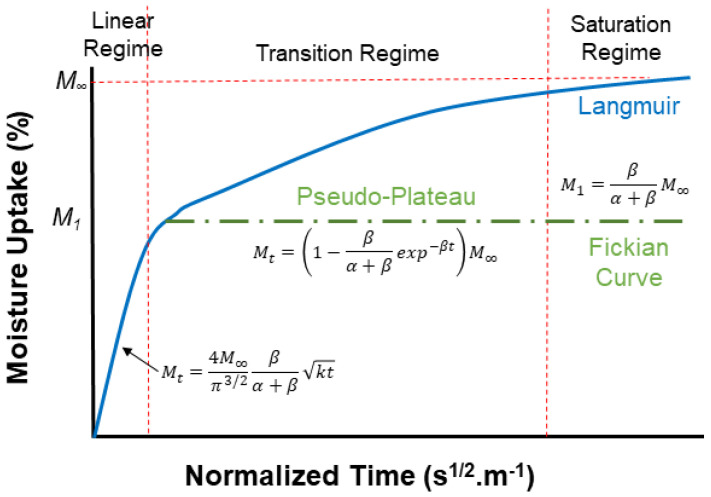
Schematic showing the three stages representative of changes in Langmuir diffusion.

This structure essentially divides the uptake regime into three stages as shown in Figure 11: (a) the initial linear regime that is similar to Fickian diffusion; (b) the longer transitional regime; and (c) the final plateau representative of attainment of equilibrium. Figure 11 differentiates between moisture levels, *M*_1_ and *M*_∞_, representing the uptake levels at the pseudo-plateau (Fickian) level and the final equilibrium level, respectively. Simple expressions representing moisture uptake as a function of time in the first two stages are also provided for comparison.

Furthermore, Popineau et al. [220] provide approximate formulations that connect moisture uptake with time, using spatial distribution expressions as follows:

(82) $$\frac{m_{free}\left(t\right)}{M_{\infty }}=\frac{\alpha }{\alpha +\beta }\left(1-\frac{8}{\pi^{2}}e^{-\kappa t}\right)$$

and

(83) $$\frac{m_{\mathrm{bound}\;}\left(t\right)}{M_{\infty }}=\frac{\alpha \beta }{\alpha +\beta }e^{-\alpha t}\left(\frac{1}{\alpha }\left(e^{\alpha t}-1\right)-\frac{8}{\pi^{2}\kappa }\left(e^{-\kappa t}-1\right)\right)$$

Following [184,251], Equation (81) can be further simplified for short times as follows:

(84) $$M_{t}=M_{\infty }\left[\left(\frac{\alpha }{\alpha +\beta }\right)\sqrt{\frac{16Dt}{h^{2}\pi^{2}}}\right],\;t\leq 0.7/\kappa$$

and for prolonged exposure times as follows:

(85) $$M_{t}=M_{\infty }\left[1-\left(\frac{\beta }{\alpha +\beta }\right)\exp\left(-\alpha t\right)\right],\;t\gg 1/\kappa$$

The characterization of pseudo-equilibrium, denoted as *M_ps_*_∞_, can be characterized as follows:

(86) $$M_{ps\infty }=M_{\infty }\left(\frac{\alpha }{\alpha +\beta }\right)$$

α can be calculated through differentiation of Equation (79), and subsequently, β can be derived using experimental data on mass uptake with time, where [252]

(87) $$-{\left(\frac{dM_{t}}{dt}\right)}^{-1}\frac{d^{2}M_{t}}{dt^{2}}\approx \;constant\;=\alpha$$

(88) $$\exp\left(-\alpha t\right)\left[\alpha {\left(\frac{dM_{t}}{dt}\right)}^{-1}M_{t}+1\right]\approx \;constant\;=1+\alpha /\beta$$

This state of pseudo-equilibrium signifies the fraction of mobile water within the sorbent and becomes noticeable as a plateau under conditions of sufficiently elevated temperatures and low specimen thicknesses, where the assumption *α*, *β* << *κ* holds true.

Among the models discussed for non-Fickian diffusion in resins, the Langmuir-type model has been reported to demonstrate good correspondence to uptake data [124,198,220,229,248,250,252,253,254,255,256,257]. Glaskova et al. [250] evaluated the performance of several models for a selected commercial epoxy resin and found that both the Langmuir-type and relaxation models yielded results that matched the experimental data. Similarly, other researchers [220,252,257] reported that Langmuir-type models produced highly consistent results and accurately predicted further mass increases based on collected experimental data. Nevertheless, there still exists a high level of concern related to accurately determining *α* and *β* that accurately correspond with levels of bound and free water in the material [8]. Various algorithms for curve fitting have been employed to determine values for *D*, *α*, *β*, and even *M*_∞_. As an example, a commonly used method involves selecting a moisture content from an apparent pseudo-equilibrium plateau in the empirical moisture sorption curve to define *M_ps_*_∞._ This enables the determination of the ratio of α to *α* + *β* from Equation (86)]. Employing an exponential curve fit based on Equation (85) yields *β*, facilitating the calculation of α using Equation (86) [220]. The slope of the initial uptake curve of *M_t_* plotted against *t*^1/2^ for times less than 0.7/κ is then utilized to ascertain the diffusion coefficient, *D*, through Equation (84) [220].

Li et al. [258], on the other hand, used a curve-fitting approach to determine the diffusion coefficient by fitting the simplified solution to Fick’s Law, as outlined in Equation (24), and subsequently constrained *D* in Equation (79) to this determined value [258]. Following this, *M*_∞_, *α*, and *β* are obtained through a curve-fitting algorithm. If *β* is assumed to be 0, it suggests no conversion, and consequently, the diffusion coefficient would be equivalent to Fickian diffusion. However, if *β* is greater than 0, water has the potential to convert from free to bound, and vice versa, allowing for a higher maximum moisture content in the sorbent compared to Fickian diffusion. It should, however, be mentioned that these fits can, at times, result in negative values of *α* and/or *β*, which would indicate invalidity of the model since *α* and *β* are probabilities of change in state of water, and hence positive.

To describe the anomalous absorption behavior, where the diffusion rate undergoes temporal changes in composite materials, a time-varying Langmuir diffusion model was proposed by Yu and Xing [259]. This model incorporates an equivalent time parameter, t*, replacing the physical time *t*, to establish the relationship between the variation rate of the diffusion coefficient and time, such that

(89) $$D=D_{0}e^{-\lambda t}$$

(90) $$t^{*}=\frac{1-e^{-\lambda t}}{\lambda }$$

where *D*_0_ is the initial diffusion coefficient, while λ is the rate at which the moisture diffusion coefficient changes over time. Thus, the time varying Langmuir diffusion model can be described as follows:

(91) $$\frac{M_{t}}{M_{\infty }}=\frac{\alpha }{\left(\alpha +\beta \right)}e^{-\beta t^{*}}y\left(t^{*}\right)-\frac{\alpha }{\left(\alpha +\beta \right)}\left(e^{-\alpha t^{*}}-e^{-\beta t^{*}}\right)+1-e^{-\alpha t^{*}}$$

where

(92) $$y\left(t^{*}\right)=1-\frac{8}{\pi^{2}}\sum_{n=0}^{\infty }\frac{1}{{\left(2n+1\right)}^{2}}e^{-\kappa {\left(2n+1\right)}^{2}t^{*}}$$

and

(93) $$\kappa =\pi^{2}D_{L}/l^{2}$$

where *D_L_* represents the diffusion obtained from the Langmuir diffusion model at the initial time, *α* signifies the bounding rate of molecules in the free phase, and *β* denotes the transition rate of molecules from the bound to the free state.

While Langmuir-type models address mechanism changes through the state of water and can be used to assess the effects of non-negligible volume changes in materials resulting from uptake and diffusion, they do not explicitly account for combinations of short-term and long-term effects, the former of which is diffusion-dominated, whereas the latter could be affected by polymer relaxation. A two-stage diffusion pattern, with the initial stage being primarily characterized by Fickian diffusion, succeeded by a gradual relaxation of the polymer, was proposed by Bagley and Long [188]. This two-stage phenomenon initially developed for cellulose acetate, is applicable to various polymer-penetrant systems, including moisture absorption in thermoset resins and composites. This model identifies two distinct stages: an initial rapid diffusion phase driven by concentration gradients, following Fickian behavior, and a subsequent phase characterized by slow polymer relaxation, allowing for additional absorption, approaching to a true final equilibrium. This latter process involves the rearrangement of polymer chains over time due to the presence of moisture penetrant molecules, resulting in further absorption at a slower rate than the initial uptake. This model can be viewed as a modified Fickian model, incorporating a time-dependent maximum moisture content, as the polymer undergoes a gradual relaxation process allowing for increased moisture sorption over time [207]. Long and Richman [157] further refined the model by including an exponential dependence on time in the consideration of surface concentration [157].

The non-dimensional form of an equation is particularly advantageous because it excludes any dimensional variable properties, making it especially useful for managing more complex systems influenced by multiple dimensional variables, and can potentially provide a clearer description of absorption behavior, revealing underlying principles. The non-dimensional form of the Langmuir-type diffusion model was used to derive an approximate solution for the three-dimensional hindered diffusion model (HDM) resulting in the dimensionless form [184,260,261]:

(94) $$D_{x}^{*}\frac{\partial^{2}n^{*}}{\partial {\left(x^{*}\right)}^{2}}+D_{y}^{*}\frac{\partial^{2}n^{*}}{\partial {\left(y^{*}\right)}^{2}}+D_{z}^{*}\frac{\partial^{2}n^{*}}{\partial {\left(z^{*}\right)}^{2}}=\mu \frac{\partial n^{*}}{\partial t^{*}}+\left(1-\mu \right)\left(n^{*}-N^{*}\right)$$

where

(95) $$n^{*}=\frac{n\left(t\right)}{n_{\infty }}\;N^{*}=\frac{N\left(t\right)}{N_{\infty }}\;t^{*}=\beta t$$

(96) $$x^{*}=\frac{x}{l}\;y^{*}=\frac{y}{w}\;z^{*}=\frac{z}{h}$$

(97) $$D_{x}^{*}=\frac{D_{x}}{l^{2}\left(\gamma +\beta \right)}\;D_{y}^{*}=\frac{D_{y}}{w^{2}\left(\gamma +\beta \right)}\;D_{z}^{*}=\frac{D_{z}}{h^{2}\left(\gamma +\beta \right)}\;\mu =\frac{\beta }{\gamma +\beta }$$

In the above, n^*^, N^*^, and t^*^ are dimensionless concentration of diffusing molecules at time *t*, the dimensionless reference concentration, and dimensionless time, respectively. Parameters x^*^, y^*^, and z^*^ are the dimensionless *x*-coordinate, *y*-coordinate, and *z*-coordinate, respectively. $D_{x}^{*}$, $D_{y}^{*}$, and $D_{z}^{*}$ are dimensionless diffusion coefficients in the *x-*, *y-*, and *z*-directions, respectively. Meanwhile, *β* and *γ* represent the probability of a bound molecule transitioning to a free state and a free molecule becoming bound, and *l*, *w*, and *h* are the length, width, and thickness, respectively. The analytical solution of the 3-D HDM can be restated as follows [125,184,244]:

(98) $$M^{*}=1-\frac{512\mu }{\pi^{6}}\sum_{P=0}^{\infty }\sum_{Q=0\;k=0}^{\infty }\sum^{\infty }\frac{1}{{\left(2P+1\right)}^{2}{\left(2Q+1\right)}^{2}{\left(2R+1\right)}^{2}}e^{-\alpha t^{*}}-\left(1-\mu \right)e^{-t^{*}}$$

where

(99) $$\alpha =\left(A_{1}{\left(2P+1\right)}^{2}+A_{2}{\left(2Q+1\right)}^{2}A_{3}{\left(2R+1\right)}^{2}\right)$$

(100) $$M^{*}=\frac{M\left(t\right)}{M_{x}}\;t^{*}=\beta t\;\mu =\frac{\beta }{\gamma +\beta }$$

and

(101) $$A_{1}=\frac{\pi^{2}D_{x}}{\beta l^{2}}\;A_{2}=\frac{\pi^{2}D_{y}}{\beta w^{2}}\;A_{3}=\frac{\pi^{2}D_{z}}{\beta h^{2}}$$

At the simplest level, assuming a linear relationship between weight gain and time suggests representing saturation concentration as a linear function of time as well, such that

(102) $$c_{s}=c_{0}+k\sqrt{t}$$

where *c_s_* is saturation concentration and *c*_0_ is the initial moisture concentration. Thus, the weight gain for a specimen of thickness *h* is given as follows:

(103) $$M=\int_{-h}^{h}cdx=2hc_{s}=2hc_{0}+2hk\sqrt{t}=M_{0}\left(1+k^{\prime }\sqrt{t}\right)$$

Given that the initial diffusion follows Fick’s law, the moisture uptake over the entire experimental time scale can be approximated following [65] as follows:

(104) $$M_{t}=M_{0\infty }\cdot \left[1+k\sqrt{t}\right]\cdot \left\{1-\frac{8}{\pi^{2}}\sum_{n=0}^{\infty }\frac{1}{{\left(2n+1\right)}^{2}}\exp\left[-\frac{Dt}{h^{2}}\pi^{2}{\left(2n+1\right)}^{2}\right]\right\}$$

where *M_t_* represents the water uptake at time t, *M*_∞0_ is the level of quasi-equilibrium water uptake, *k* is a constant related to the relaxation of the polymer structure in the second diffusion stage, *D* is the diffusion coefficient, and *h* is the thickness of the specimen.

Using the Shen and Springer [133] approximation,

(105) $$M_{t}=M_{0\infty }\left(1+k\sqrt{t}\right)\left(1-\exp\left[-7.3{\left(\frac{Dt}{h^{2}}\right)}^{0.75}\right]\right)$$

where *M*_0__∞_ represents the pseudo-equilibrium moisture content, and *k* is designated as a “relaxation coefficient” [207]. In the second phase, this relaxation is characterized by an asymptote that forms an increasing linear relationship with *t*^1/2^ instead of a constant *M*_0__∞_. Similar patterns have been observed where the asymptote takes the form of a decreasing linear function of *t*^1/2^. A declining linear asymptotic pattern, indicating the attainment of a maximum moisture content followed by subsequent weight loss, may suggest sorbent degradation, which can be identified through permanent weight loss after redrying. Thus, *k* may signify either relaxation or degradation phenomena with a simple change in sign and is thus representative of structural modification rather just relaxation.

**Figure 12:**
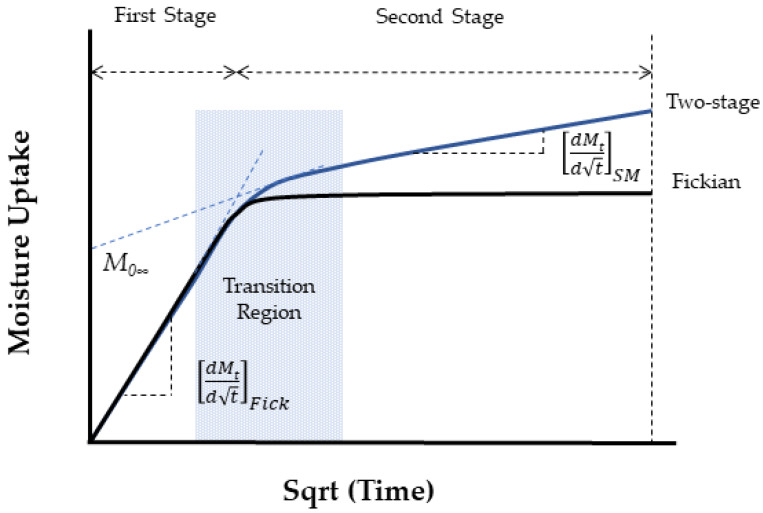
Schematic of moisture uptake in a two-stage Fickian model.

It is emphasized that the determination of *M*_0__∞_ is not a straightforward task and goes beyond merely assigning a maximum moisture content since *M*_0__∞_ specifically denotes the equilibrium moisture content related to the Fickian component within Equation (104). Consequently, the sorption pattern of *M_t_* versus *t*^1/2^ needs to be categorized into three distinct sets. As shown in Figure 12 in these sets, Fickian diffusion and structural modification play dominant roles in influencing sorption during early and later times, respectively. The remaining phase characterizes an intermediate transition region. This approach offers a comprehensive perspective on the complex interplay of Fickian diffusion and structural modifications throughout various stages of sorption.

Conducting linear fits on both sets enables the determination of *M*_0__∞_, *k*, and *D*. The ideal fit line for the Fickian-dominated phase is expected to intersect at the origin. *M*_0__∞,_ representing the pseudo-equilibrium moisture content, is obtained from the intercept of the best fit line that characterizes the gravimetric data dominated by structural modification. Once *M*_0__∞_ is established, the values for *D* and *k* can be determined from the slopes of the respective line fits as follows:

(106) $${\left[\frac{\partial M_{t}}{\partial \sqrt{t}}\right]}_{Fick}=\frac{4M_{0\infty }}{h}\cdot \sqrt{\frac{D}{\pi }}$$

(107) $${\left[\frac{\partial M_{t}}{\partial \sqrt{t}}\right]}_{SM}=M_{0\infty }k$$

where the term with the subscript SM indicates the influence of structural modification.

In the context of the two-phase Fickian model, there exists an intermediate time where *D*_2_ can be assessed. This is grounded in the assumption that the first phase has attained an equilibrium moisture content, while the second phase is still in the initial phases of sorption. Specifically, during this intermediate time, Equation (60) can be approximated as follow:

(108) $$M_{t}\approx M_{1}+M_{2}\left[\frac{4}{h}\sqrt{\frac{D_{2}t}{\pi }}\right]$$

Similarly, the model incorporating structural modification can be applied to later time intervals. In this phase, the Fickian component has attained its equilibrium moisture content, and the subsequent phase undergoes modification as dictated by the parameter *k*. Specifically, in this later timeframe, Equation (104) can be approximated as follows:

(109) $$M_{t}\approx M_{\infty }+M_{0\infty }\sqrt{t}$$

From Equations (108) and (109),

(110) $$M_{0\infty }\approx M_{1}$$

and

(111) $$M_{0\infty }k\approx \frac{4M_{2}}{h}\sqrt{\frac{D_{2}}{\pi }}$$

By employing the simplified model for structural modification, the diffusion coefficient of the second phase within the dual-phase Fickian model can be determined as follows:

(112) $$D_{2}\approx \pi {\left[\frac{M_{0\infty }kh}{\left(M_{\infty }-M_{0\infty }\right)4}\right]}^{2}$$

Hassanpour and Karbhari [262] proposed a modified model using a two-phase framework that integrates diffusion and relaxation/deterioration stages, alongside a transitional phase describing the full moisture absorption in composites and explicitly accounting for deterioration and damage that could occur during both phases. These effects can be differentiated based on discrepancies between “ideal” and “actual” diffusion and relaxation/deterioration coefficients; furthermore, the relaxation and diffusion coefficients are adjusted with modification factors *L*_1_ and *L*_2_ to effectively capture the changes. Moisture uptake in the composite can then be described as follows:

M_t_ = (Uptake due to diffusion-dominated regime) + (Uptake due to relaxation and longer-term deterioration) + (Uptake in the transitional regimes) (113)

i.e.,

(114) MtMtrans=1+L1kr1+λab+ac1−2Vfπ1−Vf2t1−exp⁡−7.3L2Dr1+λab+ac1−2Vfπ1−Vf2h2t0.75

where *M_trans_* is the equilibrium moisture uptake level, *M_t_* is the moisture uptake at time *t*, *b* and *c* represent the planar dimensions of the moisture uptake specimen, *a* is the thickness, and *λ* = 0.54 following Starink et al. [187]. *D_r_* and *D_f_* refer to the diffusion and relaxation coefficient in the resin, respectively, *L*_1_ and *L*_2_ are damage terms, and *V_f_* is the fiber volume fraction. The modification factors indicate the changes in relaxation and diffusion characteristics beyond what would be expected if a direct transition from resin to composite response, based solely on fiber volume fraction, were feasible. This approach evaluates the changes at the interface and in the resin caused by temperature, distinguishing them from the characteristics of the bulk resin, as well as the bulk resin modified by fiber volume fraction, and offers a more comprehensive understanding of the local response, and can be rewritten as follows:

(115) $$\;M_{t}=M_{trans}\left(1-\exp\left[-7.3{\left(\frac{D_{eff}L_{2}t}{h^{2}}\right)}^{0.75}\right]\right)+\;{\;M}_{trans}k_{eff}L_{1}\sqrt{t}{\;+\;M}_{trans}{\;(k}_{eff}L_{1}\sqrt{t}\;)(-\exp\left[-7.3{\left(\frac{D_{eff}L_{2}t}{h^{2}}\right)}^{0.75}\right])$$

where *D_eff_* and *k_eff_* are the effective diffusion and relaxation/deterioration coefficients, modified through correction factors as in Equation (56), with *h* representing the thickness.

Based on this analysis, it is reasonable to propose that the structural modification factor, denoted as *k*, is intricately linked to the uptake of a secondary phase. The diffusion process within this secondary phase progresses at a slower time scale, posing challenges in precisely determining the secondary equilibrium moisture content within the time constraints of most experimental investigations [69,126,159,207,221,235,249,263,264,265,266,267,268].

**Figure 13:**
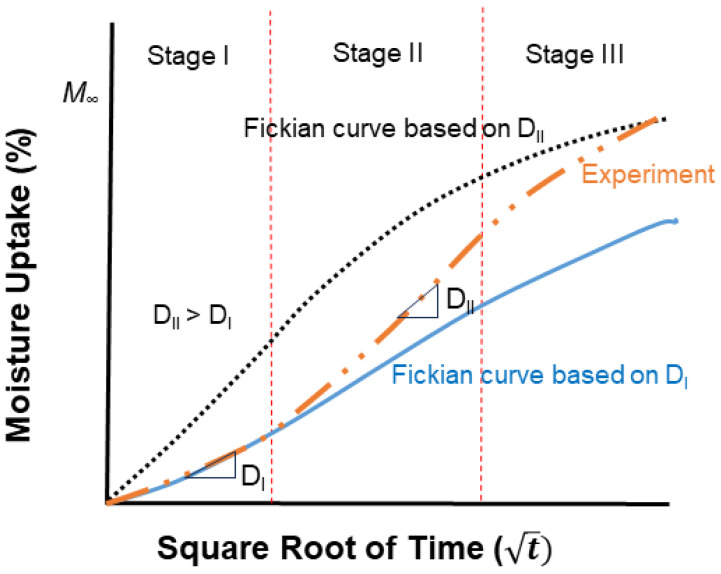
Schematic showing three stages of uptake and bounding curves following [162].

Berens and Hopfenberg [159] suggested use of a linear combination of distinct contributions from Fickian diffusion and polymeric relaxation, considered independently. The Behrens–Hopfenberg model [159] is founded upon the relaxation phenomena that occur as a result of polymer swelling during diffusion. In a glassy polymer, the diffusion of water is facilitated by Fickian diffusion, alongside changes in relaxation within the polymer network as the free volume undergoes alterations [159,269]. The total amount of absorption at time *t*, *M_t_*, can be expressed as follows:

(116) $$M_{t}=M_{F,t}+M_{R,t}$$

where *M_t_* is the moisture uptake at time *t*, *M_F_*_,*t*_ denotes the moisture uptake for the Fickian diffusion stage and *M_R_*_,*t*_ represents the moisture uptake corresponding to the relaxation- dominant stage. After a period of diffusion primarily governed by Fick’s law, swelling of the polymer leads to a gradual and significant increase in moisture sorption due to volume expansion. Simultaneously, physical or chemical degradation processes, such as polymer hydrolysis, chain breakage, the formation of small molecules, and their extraction from the composite, also take place, resulting in mass loss of the composite. If the mass loss resulting from physical or chemical degradation surpasses the increase in moisture due to swelling, it leads to a gradual reduction in moisture sorption, and vice versa. This phenomenon, encompassing swelling and initial chemical reactions, is referred to as the relaxation-dominated process and is assumed to follow first-order kinetics [159,270], such that

(117) $$\frac{dM_{R,t}}{dt}=k\left(M_{R,\infty }-M_{R,t}\right)$$

where *k* stands for the relaxation rate constant, *M_R_*_,∞_ denotes the equilibrium uptake level related to the relaxation process and *M_R_*_,*t*_ is the moisture uptake corresponding to the relaxation-dominant stage. On this basis, Berens and Hopfenberg [270] introduced a more comprehensive expression to incorporate multiple viscoelastic processes in the following form:

(118) $$M_{R,t}=\sum_{i=1}^{N}\left(M_{R_{i},\infty }\right)\left(1-e^{-k_{i}t}\right)$$

leading to

(119) $$M_{t}=M_{F,t}+M_{R,t}=M_{F,\infty }\left(1-\exp\left[-7.3{\left(\frac{Dt}{l^{2}}\right)}^{0.75}\right]\right)+M_{R,\infty }\left(1-\exp\left(-kt^{2}\right)\right)$$

where *M_F_* represents the amount of absorbed water attributed to the Fickian diffusion process, *M_R_* corresponds to the amount of absorbed water linked to relaxation phenomena, *M_F_*_,∞_ denotes the saturation level of water absorption attained in the first stage disregarding stress relaxation, *M_R_*_,∞_ represents the equilibrium moisture uptake amount related directly to the relaxation process, and *D* is the diffusion coefficient. Studies on absorption in polymers indicate that the rate-controlling mechanism depends on the size of the penetrant [238]. If absorption starts rapidly but slows down in the later stages, it suggests that diffusion exceeds relaxation. However, when these processes occur simultaneously, differentiating them based on the absorption curve becomes challenging. For a more comprehensive understanding of diffusion behavior, a modified diffusion model was introduced by Du et al. [162], outlining three distinct stages of diffusion as shown schematically in Figure 13.

In this model, stage I is primarily governed by concentration gradients, while stage II is characterized by polymer relaxation. Stage III, on the other hand, is mainly affected by aging temperatures [162], such that

(120) $$M\left(t\right)=M_{Ι}\left(t\right)+M_{\mathrm{II}}\left(t\right)+M_{\mathrm{III}}\left(t\right)$$

where *M*(*t*) is the moisture content at time *t*, *M*_I_(*t*), *M*_II_(*t*), and *M*_III_(*t*) represent the moisture contents over time in stage I, stage II, and stage III, respectively, and can be determined as follows:

(121) $$M_{I}\left(t\right)=M_{m,I}\left\{1-\exp\left[-7.3{\left(\frac{D_{I}{\;t}_{I}}{h^{2}}\right)}^{0.75}\right]\right\}$$

(122) $$M_{\mathrm{II}}\left(t\right)=M_{m,\mathrm{II}}\left\{1-\exp\left[-7.3{\left(\frac{D_{\mathrm{II}}{\;t}_{\mathrm{II}}}{h^{2}}\right)}^{\phi }\right]\right\}$$

(123) $$M_{\mathrm{III}}\left(t\right)=M_{\infty }\left\{1-\exp\left[-{\left(\alpha \left\langle t_{\mathrm{III}}-t_{\mathrm{II}}^{\infty }\right\rangle \right)}^{\beta }\right]\right\}+\varphi \left(T\right)$$

*M_m_*_,I_ and *M_m_*_,II_ represent the moisture content accrued in stage I and stage II, respectively, while *M*_∞_ is the saturated moisture content. Similarly, *D*_I_ and *D*_II_ are the diffusion coefficient for stage I and stage II, respectively. Additionally, *t*_I_, *t*_II_, and *t*_III_ denote the moisture absorption time for each stage, while $t_{\mathrm{II}}^{\infty }$ signifies the maximum absorption time at stage II. Parameters ϕ, α, and β remain to be determined experimentally, and *h* denotes the thickness. The Macaulay bracket, < >, used for the time delay term, *t*_III_ − $t_{\mathrm{II}}^{\infty }$, signifies that third stage behavior occurs only when *t*_III_ is greater than or equal to $t_{\mathrm{II}}^{\infty }$. Additionally, $\varphi \left(T\right)$ functions as a dependency on aging temperature and is expressed as follows:

(124) $$\varphi \left(T\right)=a\left\{1-\left[\exp\left(-k\left(T-T_{room\;}\right)\right)\right]\right\}$$

where a and *k* denote parameters to be determined through experiments, with *T* representing temperature, and *T_room_* indicating room temperature.

Another model, proposed by Gavril’eva et al. [271], introduces an additive model of moisture diffusion with a time-dependent diffusion coefficient and relaxational boundary conditions to address the challenges in achieving agreement between experimental and calculated results, emphasizing complex interactions of factors affecting moisture absorption kinetics in fiber-reinforced polymer composites. These factors involve redistribution of free volume, plasticization effect of water on the composite, damage formation, changes in stress concentration distribution, anisotropy, and swelling. It is difficult to develop integrated mechanistic models for each of these. In contrast, developing additive models that incorporate multiple processes is entirely feasible. The time-dependent nature of diffusion is expressed as follows:

(125) $$D\left(t\right)=D_{0}h\left(t\right)$$

where *D*_0_ represents the initial diffusion coefficient, which remains constant, the function *h*(*t*) denotes the time dependence of the diffusion coefficient, with *h*(*t*) being greater than zero, and *t* signifies the time variable. By introducing a new time scale as *τ*

(126) $$d\tau =h(t)dt,\;\mathrm{where}\;\tau =\int_{0}^{t}h\left(t\right)\;dt$$

a one-dimensional diffusion equation can be obtained [49,243]:

(127) $$\frac{dc\left(x,\tau \right)}{d\tau }=D_{0}\frac{d^{2}c\left(x,\tau \right)}{d^{2}x}$$

where *τ* is time, and the concentration of diffusing material at a point with coordinate *x* and time *τ* is denoted by *c*(*x*,*τ*), with *D*_0_ representing the initial diffusion coefficient. In the new time scale, *τ*, the assumption is made that the boundary condition for Equation (127) takes the following form:

(128) $$\mu \left(\tau \right)=\mu_{1}+\left(\mu_{0}-\mu_{1}\right)e^{-r\tau }$$

where parameters *μ*_0_ and *μ*_1_ denote the initial and limiting solubility, respectively, while *r* represents the relaxation constant (*r* ≥ 0). Equation (128) defines the boundary condition, illustrating the structural relaxation of the polymer binder influenced by moisture [271]. Moisture absorbed is then determined by measuring the mass of body at a specific time, *τ*, and is represented as follows:

(129) $$M\left(t\right)=\hat{M}\left(\tau \right)=\int_{0}^{t}c\left(x,\tau \right)\;dx$$

such that the quantity of moisture absorbed at time by an initially dry plate is as follows [272]:

(130) $$M\left(\tau \right)=M_{1}+\left(M_{0}-M_{1}\right)e^{-r\int_{0}^{t}h\left(t\right)dt}-8\sum_{k=0}^{\infty }S_{k}$$

where *M*_0_ and *M*_1_ represent the limiting moisture content before, and after, stress relaxation due to moisture absorption in a plate with thickness *l*. *r* is the relaxation constant, and *h*(*t*) denotes the time dependence of the diffusion coefficient. The term *S_k_* is expressed as follows:

(131) $$S_{k}=\frac{\left(M_{0}n_{k}^{2}D_{0}/l^{2}-M_{1}r\right)e^{-n_{k}^{2}D_{0}/{l\;}^{2}\int_{0}^{t}h\left(t\right)dt}+r\left(M_{1}-M_{0}\right)e^{-r\int_{0}^{t}h\left(t\right)dt}}{n_{k}^{2}\left(n_{k}^{2}D_{0}/l^{2}-r\right)}$$

where *l* is the thickness of the plate, and *n_k_ =* 2*π*(2*k* + 1). The model incorporates relaxation with Fickian diffusion, and when the relaxation term, *r*, is zero, the structure is basically that of Fickian diffusion.

Another version of the Fickian model, proposed by Upadhyay and Mishra [273], accounts for the impact of temperature on moisture absorption. In this equation, modifications have been proposed through the introduction of empirical parameters into the one-dimensional Fickian solution itself. To account for the concentration dependency of moisture absorption, the diffusion coefficient is described in relation to the instantaneous moisture concentration as follows:

(132) $$D_{eff}(t)=\;D[1+d\cdot M^{2}(t)]$$

where *D* is the coefficient of diffusion in a moisture-free condition, while *d*, a constant determined empirically, denotes the moisture sensitivity of the diffusion coefficient. It should be noted that when materials are exposed to moisture, not all water molecules in the environment are free; rather, they are weakly hydrogen-bonded. Therefore, they are not readily available for diffusion. A molecule cannot diffuse until it detaches from surrounding molecules. Consequently, the environmental moisture concentration is reduced by a factor, denoted as *b*, such that

(133) $$M_{m\;eff}={b\cdot M}_{m\;}$$

where *M_m_* stands for the apparent atmospheric moisture concentration, and *b* represents a dimensionless constant, with its value falling between 0 and 1. Unlike the classical assumption, that assumes the composite can absorb moisture to its full capacity instantaneously, in reality, a finite amount of time is always required to attain the boundary conditions, which refers to the time taken to account for time delays and lags between the actual process and the idealized assumption of instantaneous boundary conditions. This discrepancy implies that the actual time taken to reach a given concentration level within the material will always exceed the classical model’s predictions. This aspect can be considered by introducing a time-shift factor *t_s_*, such that

(134) $$t_{\;eff}={(\;t-t}_{s\;}),\;t_{s\;}<t$$

where the variable *t_s_* indicates the duration necessary for the establishment of the boundary condition within the material. Elevated temperatures result in increased kinetic energy and mobility of molecules, leading to higher coefficients of moisture diffusion. Considering the temperature dependency of the diffusion coefficient, the coefficient of diffusion can be formulated as follows:

(135) $$D(T)=D_{R}\exp\left(\frac{E}{KT_{R}}-\frac{E}{KT}\right)$$

where *D*(*T*) represents the diffusion coefficient at absolute temperature *T*, *D_R_* denotes the diffusion coefficient at absolute reference temperature *T_R_*, *E* represents a constant (activation energy for the process), and *K* is the Boltzmann constant, with a value of 1.38 × 10^−23^ joules per kelvin (J/K). Using Equations (133)–(135), Upadhyay and Mishra [273] concluded that moisture content can be determined as follows:

(136) $$M\left(t\right)=\frac{2}{\sqrt{\pi }}\cdot b\cdot \left(M_{m}\right)\cdot {\left[\frac{D_{R}\exp\left(\frac{A}{T_{R}}-\frac{A}{T}\right)\cdot \left(t-t_{s}\right)}{L^{2}}\right]}^{1/2}$$

where *M*(*t*) represents the moisture uptake at time *t*, *M_m_* is the apparent atmospheric moisture concentration, *b* is a dimensionless constant, *D_R_* denotes the coefficient of diffusion at the absolute reference temperature *T_R_*, *T* represents the absolute temperature, *L* is the thickness of the specimen, and *t_s_* measures the time required for the establishment of the boundary concentration in the material. Furthermore, *A* is another constant replaced by *E/K*, for simplicity.

As previously mentioned, with increasing temperature or humidity, Fickian diffusion mechanisms lose dominance, enabling other mechanisms to play a more significant role in moisture transport. To model concurrent diffusive, relaxation, and chemical degradation sorption or damage mechanisms, Xin et al. [196] considered the contribution of moisture absorbed by each as superimposable with the total moisture sorption *M_t_*, divided into a diffusion-dominated uptake *M_F,t_*, a polymer relaxation-dominated uptake *M_R,t_*, and a composite damage-dominated uptake *M_D,t_*, expressed as follows:

(137) $$M_{t}=M_{F,t}+M_{R,t}+M_{D,t}$$

where *M_F_*_,*t*_, *M_R,t_*, and *M_D,t_* represent the individual contributions of the Fickian, relaxation, and damage processes at time t as introduced by Behrens and Hopfenberg [159], corresponding to the factors. Microcracks and/or voids can be formed at the interface [274] between the fiber and polymer or between layers/laminae as a consequence of environmental exposure resulting in chemical degradation and water pressure. All these mechanisms would potentially result in a significant, and rapid, increase in moisture uptake levels, especially over the composite damage-dominated regime, which is represented through an exponential form based on the trends seen in experimental data. The composite damage-dominated uptake, which is initiated after a specific aging time is expressed through the introduction of a Macauley bracket as follows:

(138) $$M_{D,t}=M_{D,i}e^{\Omega \left\langle t-t_{D}\right\rangle }$$

where *t_D_* represents the initial time when damage is initiated, Ω is a parameter associated with the uptake influenced by damage, *M_D,i_* is the mass sorption at the onset of damage, and ⟨ ⟩ denotes the Macauley bracket operator. Xin et al. express moisture absorption [196] as follows:

(139) $$M_{t}=M_{F,\infty }\left(1-\exp\left[-7.3{\left(\frac{Dt}{h^{2}}\right)}^{0.75}\right]\right)+\sum_{i=1}^{N}\left(M_{R,\infty }\right)\left(1-e^{-k_{i}t}\right)+M_{D,i}e^{-\Omega \left\langle t-t_{D}\right\rangle }$$

where *M_F_*_,∞_ is the saturation level of water absorption disregarding stress relaxation, *M_R,∞_* is the equilibrium moisture uptake amount related to the relaxation process, *M_D,i_* is the mass sorption at the onset of damage, *k* stands for the relaxation rate constant, and *D* is the diffusion coefficient.

A function characterized by three parameters, the equilibrium moisture content, initial diffusion coefficient, and the rate of diffusion variation over time can be employed to describe the absorption dynamics associated with non-Fickian diffusion. In contrast to the Fickian model, this approach [259] introduces an equivalent time parameter, denoted as t*, rather than the physical time, to establish the relationship between the variation rate of the diffusion coefficient and time. The diffusion coefficient *D* is extremely sensitive to temperature in the context of moisture absorption and can be empirically expressed as follows:

(140) $$D=D_{0}e^{-\lambda t}$$

(141) $$t^{*}=\frac{1-e^{-\lambda t}}{\lambda }$$

leading to a solution in the form of a trigonometrical series for the Fickian model:

(142) $$\frac{M_{t}}{M_{\infty }}=1-\sum_{n=0}^{\infty }\frac{8}{{\left(2n+1\right)}^{2}\pi^{2}}e^{\frac{-D\;\left(2n+1\right)\pi^{2}t^{2}}{l^{2}}}$$

where, at time *t*, *M_t_* represents the amount of moisture absorbed, *M*_∞_ is the maximum amount of moisture that can be absorbed, *n* is an integer indicating the number of terms in the series, *D* is the diffusion coefficient, and *l* is the thickness of the sample. Incorporating an equivalent parameter, t* and *D* into Equation (142), where *D*_0_ represents the initial diffusion coefficient and *λ* signifies the rate at which the moisture vapor diffusion coefficient changes over time, yields the time-varying diffusion model as follows:

(143) $$\frac{M_{t}}{M_{\infty }}=1-\sum_{n=0}^{\infty }\frac{8}{{\left(2n+1\right)}^{2}\pi^{2}}e^{\frac{-D_{0}\;\left(2n+1\right)\pi^{2}t^{*}}{l^{2}}}$$

The time-varying diffusion coefficient model can be presented by modifying the classic Fickian model, with the diffusion coefficient treated as a function of time as suggested by Weitsman [243], such that

(144) $$\frac{\partial C}{\partial t}=D_{0}\exp\left[-\frac{B}{\theta \left(t\right)}\right]\frac{\partial^{2}C}{\partial x^{2}}$$

where *D*_0_ and *B* represent material constants, while *θ*(*t*) signifies the absolute temperature, and results in the following:

(145) $$C\left(x,t\right)=C_{0}\left\{1+\frac{4}{\pi }\sum_{n=1}^{\infty }\frac{{\left(-1\right)}^{n}}{2n-1}cos\left[\frac{(2n-1)\pi x}{2L}\right]exp\left[-{\left(\frac{(2n-1)\pi }{2}\right)}^{2}t^{*}\right]\right\}$$

where

(146) $$t^{*}=\frac{D_{0}\int_{0}^{t}exp\left[-\frac{B}{\theta (s)}\right]ds}{L^{2}}$$

If the integral in Equation (145) equals *t*, diffusion is independent of time and simplifies to Fickian diffusion.

Roy et al. [171] suggested that viscoelastic effects influencing moisture absorption in polymers could be addressed by introducing a time-dependent diffusion coefficient and showed that moisture absorption can be effectively represented by incorporating a diffusion coefficient that increases exponentially to a plateau in the form of a Prony series, such that

(147) $$D\left(t\right)=D_{0}+\sum_{r}D_{r}\left[1-\exp\left(-\frac{t}{\tau_{r}}\right)\right]$$

leading to

(148) $$\frac{M_{t}}{M_{\infty }}=1-\frac{8}{\pi^{2}}\sum_{n=0}^{\infty }\frac{1}{{\left(2n+1\right)}^{2}}\exp\left\{\frac{-{\left(2n+1\right)}^{2}\pi^{2}}{l^{2}}\times \left[D_{0}t+\sum_{r=1}^{R}D_{r}\left[t+\tau_{r}\left(e^{-\frac{t}{\tau_{r}}}-1\right)\right]\right]\right\}$$

where the primary factors, *M*_∞_, *D*_0_, *D_r_*, and *τ_r_*, are determined through the process of fitting equation to the experimental data. *M_t_* denotes the total amount of diffusing substance that has entered the material at time *t*, and *M*_∞_ is the corresponding quantity after infinite time. *D*_0_ and *D_r_* are the unknown temperature-dependent Prony coefficients, *τ_r_* is the time constant governing the time variation of *D*, corresponding to the retardation times, and *n* is the number of terms in the series.

The models discussed inherently assume that moisture uptake is based on resin characteristics and damage/defects in the composite including fiber–matrix debonding which can lead to increased moisture uptake due to wicking. Fibers cause anisotropy in the composite and thus also cause diffusion to be anisotropic with diffusion being fastest in the direction of fibers and slowest through the thickness. Fiber volume fraction, as seen through the formulation of some of the models, plays an important role both in terms of acting as a barrier to uptake and in increasing the tortuosity of the path of moisture ingress. Moisture uptake kinetics as a function of fiber volume fraction have been studied by Ray [115] and Kondo and Taki [275], who reported that diffusion kinetics were not affected. However, other studies have reported significant dependence on fiber structure with directional diffusion ratios changing based on fabric type and fiber orientation [16,140,207,276,277,278] with a typical range of 25 to 53%. Korkees [55] and Dana et al. [279] presents an extensive review of fiber structure effects on diffusion in carbon/epoxy composites, emphasizing that the moisture uptake and diffusivity are significantly influenced by factors such as fiber volume fraction, fabric architecture, layup orientation, void content, and integrity of the fiber–matrix interfacial bond. Further details on directional diffusion are also reported by Korkees et al. [200], who reported that diffusion along fibers was three times faster than across them, and seven times faster than that in the through-thickness direction, In addition, they further emphasized the need to consider a two-stage response with an initial rapid Fickian response being, followed by a much slower second-stage that could extend for over 3.7 years.

These considerations, however, assume that the fibers themselves do not absorb moisture. In the case of aramid fibers, and others with similar morphologies, models need to consider two additional factors: (a) the uptake within the fiber itself and its contribution to overall diffusional response, and (b) the effect of fiber anisotropy, which extends to uptake and hence results in axial and radial diffusivities in the fiber itself. While these are not the focus of the current review, the interested reader is referred to the work by Aronhime et al. [170], Allred and Lindross [280], Cervenka et al. [281], and Wang et al. [282].

Thus, a large number of models have been developed to address diffusion taking into account a range of mechanisms and approaches, often differentiating moisture uptake into phases, where at least one is considered to be diffusion-dominated. The choice of method depends on the details of the material and exposure condition and must be considered carefully prior to selection.

## 6. Conclusions

FRP composites used in civil, marine, and offshore infrastructure applications are exposed to a range of environmental conditions, among which those of humidity and immersion are of specific interest because of their commonality and the complexity of resulting mechanisms in the polymer and composite. Based on a focused review, the following generic conclusions can be drawn:

- Transport of moisture into a polymer composite is affected through a range of mechanisms, including through interaction with the polymer chain itself and sorption into the volume. Rates of transport can be accelerated through damage in the composite in the form of microvoids/microcracks and fiber–matrix debonding. The interactions between the sorbed moisture and the constituents of the FRP composite are complex and can change through the service life of the structure.
- Uptake of solution into a composite can result in a range of reversible and irreversible mechanisms, which are affected by factors such as exposure environments, including temperature, constituent materials, extent of cure progression, and pre-existing damage.
- The effect of humidity levels on moisture uptake is dependent not just on the level of the humidity, but also on the temperature of exposure. Although levels of equivalence are often used between saturated humidity and immersion, the effect of both on the rate of uptake and maximum uptake are significantly different.
- Moisture uptake profiles can range from the extremes of linear to exponential with sigmoidal and stepped profiles in between. These can often be characterized by diffusion coefficients and levels of moisture uptake equilibrium, both of which may have multiple phases based on the mechanics of sorption and the resulting shape of the uptake.
- Both rate coefficients and levels of equilibrium (transition, intermediate, and maximum) are affected by the temperature and humidity of exposure, as well as conditions of immersion. Increases in temperature generically increase rates of uptake through increases in molecular mobility. However, temperature increases, as well as initial exposure to moisture, can accelerate cure progression in FRP composites that have not attained complete polymerization prior to exposure, leading to a competition between phenomena that increase performance while decreasing capacity for sorption and those that increase sorption and deterioration.
- The Fickian diffusion model is most commonly used to describe moisture uptake and assumes that the diffusion coefficient is independent of moisture concentration and of its through thickness location, and that an equilibrium level of moisture uptake is attained which is independent of temperature of humidity exposure/immersion. Research has, however, shown that there are significant deviations from Fickian response, resulting in the development and use of stepped and phased models.
- In considering the appropriate model to describe the uptake response of a FRP composite, it is important to consider mechanisms of change in the constituents, as well as effects of aspects such as concentration at saturation and solvent activity. In this regard, the range of responses can be summarized below.

- Henry’s law, where

$$C_{sat}=SP$$

- Freundlich’s relation (power law), where

$$\;C_{sat}=a{\left(\frac{P}{P_{sat}}\right)}^{b}$$

- Fick’s law, where

$$C_{sat}=\frac{-\frac{J}{D}P_{sat}}{\frac{dP}{dx}}$$

- Langmuir response, where

$$C_{sat}=\frac{cP}{1+dP}$$

- Dual phased sorption, where

$$C_{sat}=SP+\frac{cP}{1+dP}$$

in which *C_sat_* is the water concentration at saturation, *S* is the solubility of water in the material (i.e., Henry’s constant), *P* is the water partial pressure, *P_sat_* is the saturation water pressure (such that $\%RH=100\frac{P}{P_{sat}}$), *J* is the amount of moisture per unit area per unit time (defined as diffusion flux), *D* is the diffusion coefficient (which is a constant) and *a*, *b*, *c*, and *d* are constants that represent solvent activity.

- The Langmuir model intrinsically relates changes to the probability of conversion between two states of water, free and bound, and thus captures changes in uptake based on the stage of moisture uptake. This addresses some of the deviations from Fickian response but does not address longer-term effects, such as relaxation.
- Phased and stepped models assume that inherent changes in the material are due to the effects of uptake catalyze/initial additional/new mechanisms as the level of uptake increases, representing the changes through steps/phases.
- While the two-phased Fickian and the two-step (structural modification) models share the basic setup characteristics of dividing uptake into three regimes with the initial being diffusion-dominated and the second being a transition, they differ in the consideration of the third, with the former positing diffusion with a contribution to the first stage through two simultaneous mechanisms of diffusion and the latter considering two distinct mechanisms, initially a faster diffusion-dominated regime, and then a slower relaxation/deterioration-dominated regime. The two-step models are thus able to better capture response when there is apparent mass loss due to leaching of lower-molecular-weight species and/or constituents of the FRP composites.

While the material provided in the review covers a range of models, it is cautioned that selection of a model depends on factors such as details of the material and its constituents, length of assessment, aspects of environmental exposure, and operative mechanisms. In many cases, experimental/field data are only available for short periods of time, thus obscuring/neglecting materials level changes that cause diffusional deviations from the simple Fickian response. It is emphasized that more research needs to be conducted to develop a comprehensive understanding not only of mechanisms and methods of describing uptake, but of directly linking these to the prediction of long-term response and durability.

## Figures and Tables

**Table 1:** Overview of degradation mechanisms in composites caused by moisture. (F = Fiber, M = Matrix, I = Interphase).

Classification of Mechanism	Degradation Mechanism	F	Location M	I	Reversible? Y/N
Chemical	Hydrolysis		×	×	N
	Pitting	×			N
	Chain Scission		×		N
	Debonding			×	N
Physical	Plasticization		×	×	Y
	Swelling		×		Y
	Leaching	×	×	×	N
	Relaxation (Physical Aging)		×		N
Physio-mechanical	Microcracking		×	×	N
	Micro voids		×	×	N

**Table 2:** Typical values of Freundlich exponents.

Material	a	b	Reference
Resin: 3501	6.3	1.7	[82]
Resin: NMD 2373	9.9	2.3	[82]
Composite: T300/1034	1.4	2	[133]
Composite: Glass/Epoxy	1	1	[139]
Composite: T300/1034	1.7	1	[146]
Composite: AS/3501-5	1.9	1	[146]
Composite: E-glass/epoxy at 20 °C	0.216	1.188	
Composite: E-glass/epoxy at 40 °C	0.496	1.752	
Composite: E-glass/epoxy at 60 °C	0.752	3.682	

## Data Availability

No new data were created or analyzed in this study. Data sharing is not applicable to this article.
